# LL‐37 Inhibits TMPRSS2‐Mediated S2' Site Cleavage and SARS‐CoV‐2 Infection but Not Omicron Variants

**DOI:** 10.1111/cpr.70060

**Published:** 2025-05-15

**Authors:** Zhenfei Bi, Wenyan Ren, Hao Zeng, Yuanyuan Zhou, Jian Liu, Zimin Chen, Xindan Zhang, Xuemei He, Guangwen Lu, Yuquan Wei, Xiawei Wei

**Affiliations:** ^1^ Laboratory of Aging Research and Cancer Drug Target, State Key Laboratory of Biotherapy, National Clinical Research Center for Geriatrics, West China Hospital Sichuan University Chengdu China; ^2^ Department of Hepatobiliary Surgery, Xinqiao Hospital Army Medical University Chongqing China; ^3^ Institute of Biomedicine and Biotechnology Shenzhen Institute of Advanced Technology, Chinese Academy of Science Shenzhen China

**Keywords:** LL‐37, omicron variants, SARS‐CoV‐2 infection, spike protein, TMPRSS2

## Abstract

Continual evolution of SARS‐CoV‐2 spike drives the emergence of Omicron variants that show increased spreading and immune evasion. Understanding how the variants orientate themselves towards host immune defence is crucial for controlling future pandemics. Herein, we demonstrate that human cathelicidin LL‐37, a crucial component of innate immunity, predominantly binds to the S2 subunit of SARS‐CoV‐2 spike protein, occupying sites where TMPRSS2 typically binds. This binding impedes TMPRSS2‐mediated priming at site S2' and subsequent membrane fusion processes. The mutation N764K within S2 subunit of Omicron variants reduces affinity for LL‐37 significantly, thereby diminishing binding capacity and inhibitory effects on membrane fusion. Moreover, the early humoral immune response enhanced by LL‐37 is observed in mice against SARS‐CoV‐2 spike but not Omicron BA.4/5 spike. These findings reveal the mechanism underlying interactions amongst LL‐37, TMPRSS2 and SARS‐CoV‐2 and VOCs, and highlight the distinct mutation for Omicron variants to evade the fusion activity inhibition by host innate immunity.

## Graphical abstract

Cathelicidin LL‐37 inhibits SARS‐CoV‐2 by blocking the catalytic activity of TMPRSS2. The N764K mutation in the spike S2 subunit of Omicron variants confers resistance to LL‐37‐induced inhibition of TMPRSS2‐mediated S2' priming.

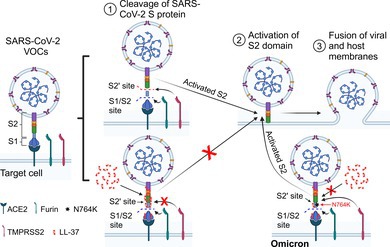

## 1 Introduction

Severe acute respiratory syndrome coronavirus 2 (SARS‐CoV‐2) contributes to the ongoing coronavirus disease 2019 (COVID‐19) pandemic, primarily due to the emergence of Omicron and its subvariants [1, 2, 3]. To bind to and fuse with the host cell, SARS‐CoV‐2 utilises the spike, an important structural homotrimeric transmembrane glycoprotein, to recognise its receptor on the surface of cells and mediate the fusion of the viral envelope with the cell membrane by sequential catalysis of host proteases [4, 5]. Also, spike is currently the primary antigenic protein that induces host adaptive immune responses. Amino acid changes in spike respond to increased spreading and robust immune evasion [6]. For example, Alpha (B.1.1.7), Beta (B.1.351), Gamma (P.1) and Omicron variants all possess the mutation N501Y, which can enhance the ACE2 affinity [7]; the mutation D614G is the turning point for SARS‐CoV‐2 evolution, as it enhances trimer opening and virus transmissibility by disruption of the D614‐K854 salt bridge, promoting the emergence of all VOCs including Omicron variants [6, 8, 9]; other mutations in the RBD domain of Omicron and its subvariants such as mutations K417N, S477N, T478K/R and E484A drive the antibody escape [10]. Continual evolution of SARS‐CoV‐2 spike drives the emergence of new variants (BQ.1, XBB, XBB.1.9, XBB.2.3 and EG.5.1), raising concerns that they may trigger extraordinary changes in the original pattern of host immune responses, leading to impaired immunity and compromise of current COVID‐19 vaccines and clinical treatment [11, 12]. It is crucial to understand how these variants orientate themselves towards the host immune defence for controlling future pandemics.

Recent evidence suggested two key proteolytic cleavage sites for spike fusion machinery: (i) The first cleavage occurs at the polybasic cleavage site (PBSC) which locates the S1 and S2 boundary (S1/S2) and is cleaved by furin‐like proteases to release the S2 subunit [13, 14]; (ii) The second cleavage occurs at the S2' site located immediately downstream of the S1/S2 and is cleaved primarily by transmembrane serine protease 2 (TMPRSS2) to expose the fusion peptide [4, 15]. If the SARS‐CoV‐2 and ACE2 complex does not encounter TMPRSS2, or the target cells express insufficient TMPRSS2, the virus may employ the suboptimal endosomal entry by cleaving the S2' site via cathepsin B/L [16, 17]. The encoded spike can be trafficked to the infected cell membrane and engage with ACE2 on the surface of neighbouring cells, leading to cell–cell fusion and production of multinuclear giant cells (also known as syncytia) [18, 19]. Spike‐mediated syncytia is closely associated with viral dissemination, immune evasion and disease progression, and represents a novel feature of severe COVID‐19 induced by SARS‐CoV‐2 [19, 20, 21]. Mutations in spike always orchestrate most of its binding energy towards fusion, such as the early described mutation D614G that renders FPPR more dynamic [8, 9], mutation P681H/R that increases its affinity to furin and augments syncytia formation [22]. More recently, mutations Q954H and N969K in the HR1 domain of the Omicron S2 subunit make core helices highly dynamic compared to other VOCs, which enables it to be better primed for fusion [6]. Although many studies have uncovered mutations in spike that link to fusion activity and host protease preference [23], or mutations outside of spike (e.g., nucleocapsid protein, Orf9b and Orf6) that inhibit RNA sensing and downregulate the expression of related interferon‐stimulated genes (ISGs) [24], the mechanism underlying how Omicron variants render the host innate immunity ineffective regarding the inhibition of its fusion activity has not been touched yet.

Innate immunity exerts strong selective pressure during SARS‐CoV‐2 infection and transmission. Notably, ISGs including CH25H, LY6E and ZMPSTE24 [25, 26, 27], and innate immune molecules including human antimicrobial peptides (AMPs) [28, 29], show anti‐SARS‐CoV‐2 activity by direct or indirect blocking fusion process. Of which, peptide LL‐37, derived from the hCAP‐18 protein encoded by *CAMP* gene, is the sole identified member of the cathelicidin family of antimicrobial peptides in humans to date [30]. The physiological concentration of LL‐37 is in the range of 2–5 μg/mL; however, the concentration can rise up to 20 μg/mL in bronchoalveolar lavage fluid (BALF) and 80 μg/mL in nasal secretions when the host is infected with pathogens [31]. Several clinical analyses have demonstrated the negative correlation of LL‐37 levels in sera with disease severity induced by SARS‐CoV‐2 [32, 33, 34], and supplementation of vitamin D (a positive regulator for *CAMP*) can alleviate the disease progression in COVID‐19 patients [35]. Given the importance of LL‐37 in respiratory innate immune defence, the underlying effects on SARS‐CoV‐2 and VOCs, especially Omicron and its subvariants, should be fully explored.

Herein, we report the role of LL‐37 in combat between the host and SARS‐CoV‐2 and VOCs. We uncover a mechanism that LL37 can remarkably inhibit the SARS‐CoV‐2‐induced membrane fusion activity with host cells and infection through binding to the spike S2 subunit and blocking TMPRSS2‐mediated S2' priming process. Furthermore, we investigate how Omicron variants escape from the LL‐37‐induced inhibition of membrane fusion and subsequent infection. Finally, we also demonstrate the effects of LL37 on humoral immunity against SARS‐CoV‐2 and Omicron variants. This study might elucidate how innate immune elements function as an inhibitor against TMPRSS2‐dependent viruses including SARS‐CoV‐2 that mediates membrane fusion, offering perspectives for specific mutations within Omicron variants to evade the fusion activity inhibition by host innate immunity.

## 2 Results

### 2.1 LL‐37 Inhibits the Cell Entry of SARS‐CoV‐2 and VOCs but Not Omicron Variants

**FIGURE 1:**
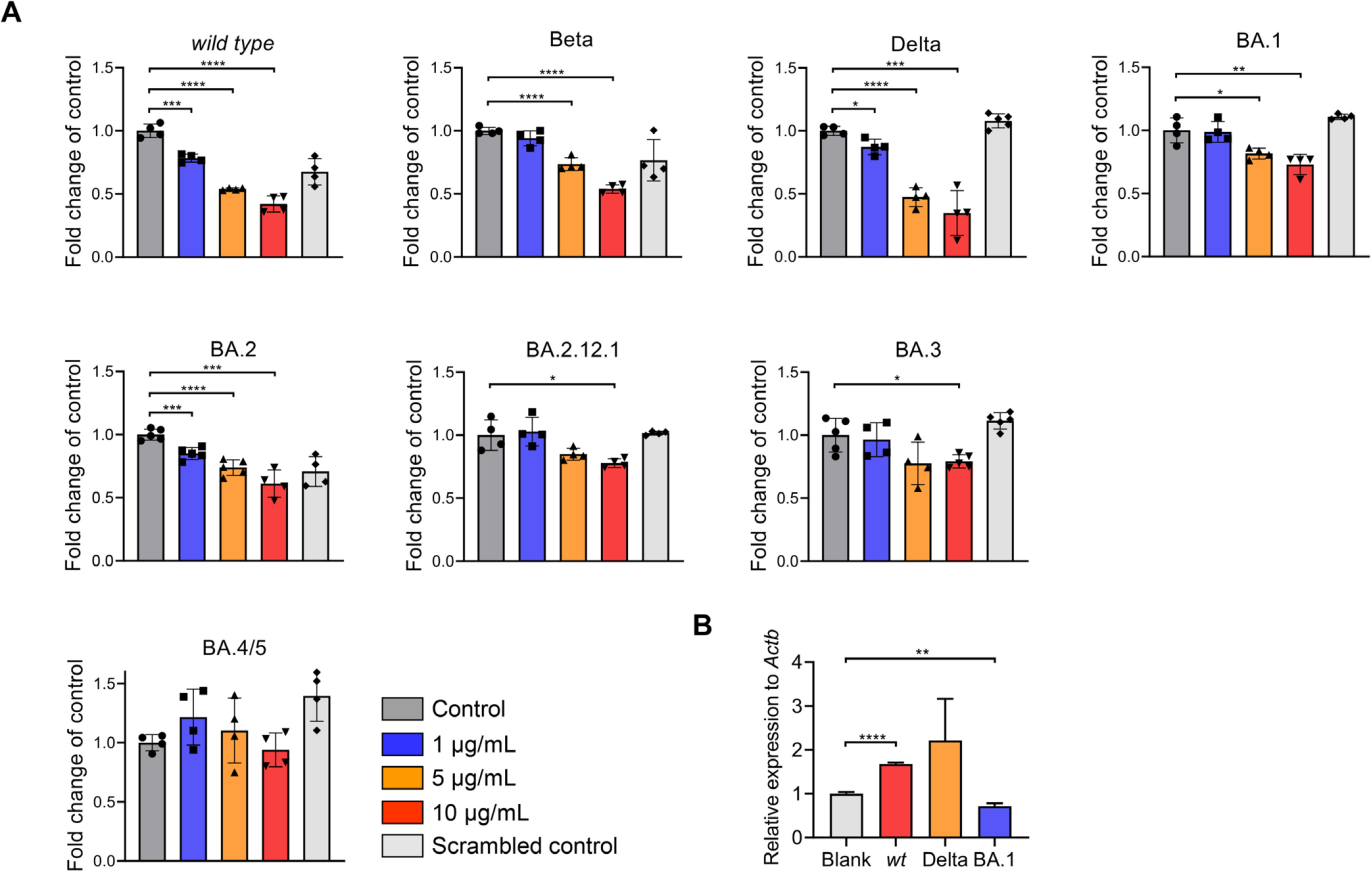
LL‐37 inhibited the entry of SARS‐CoV‐2 spike pseudoparticles and VOCs but not Omicron variants. (A) SRAS‐CoV‐2 and VOCs spike pseudoparticles were pretreated for 2 h with indicated concentrations of LL‐37, and then added to the 293T‐A2 cells. Infection rates were assessed at 2 days post infection by measuring the RLU of luciferase activity. (B) qPCR analysis of mRNA levels of *Camp* in lungs of transgenic human *ACE2* mice which were intranasally instilled with SARS‐CoV‐2 and VOCs of Delta and Omicron BA.1 respectively. Data represent the mean ± SEM. Significance is indicated by *p ≤ 0.05, **p ≤ 0.01, ***p ≤ 0.001, ****p ≤ 0.0001.

We previously investigated the enzymes or cationic peptides in respiratory tracts that are involved in SARS‐CoV‐2 infection, such as trypsin‐like enzymes, cathepsins and AMPs. Notably, we showed LL‐37, a cationic cathelicidin with an overall positive net charge (+6), could significantly inhibit the entry of SARS‐CoV‐2 and VOCs spike pseudovirus including Beta and Delta variants (Figures 1A and S1), coinciding with recent studies [34, 36, 37]. Nevertheless, we firstly uncovered that the inhibitory effects were compromised against Omicron variants including BA.1, BA.2, BA.2.12.1, BA.3 and BA.4/5 spike pseudovirus (Figures 1A and S1). In addition, we intranasally instilled mice with original SARS‐CoV‐2, and VOCs of Delta or Omicron BA.1 respectively, and harvested lung tissues for gene detection. The LL‐37 expression level was significantly upregulated following infection with original SARS‐CoV‐2 or Delta variant but not Omicron BA.1 compared with that of vehicle control on 3‐day post infection (dpi) (Figure 1B), the time which is reported as the peak viral loads after infection with SARS‐CoV‐2 for mice [38, 39, 40], corresponding to the compromised inhibition of Omicron variants by LL‐37.

To understand the LL‐37 transcriptional map in respiratory tracts when infected with SARS‐CoV‐2, we analysed the LL‐37 expression in BALF for the first time. The comparison revealed higher expression of LL‐37 in macrophages, epithelial cells, and, in general, lower expression in DC and T cells (Figure S2A). LL‐37 expression was minimum in patients with severe COVID‐19 compared with that of the normal or moderate patients (Figure S2B). Further, we enquired which type of cells SARS‐CoV‐2 affects the LL‐37 expression in. As shown in Figure S2C, SARS‐CoV‐2 infection downregulated the LL‐37 expression in all cell types; notably, macrophages from the normal, moderate to severe patients showed a gradient descent of LL‐37 expression, suggesting that the COVID‐19 severity was inversely proportional to the respiratory level of LL‐37.

### 2.2 LL‐37 Inhibits the SARS‐COV‐2‐Induced Member Fusion and Is Not Concerned With Blocking of RBD Domain or ACE2 Receptor

**FIGURE 2:**
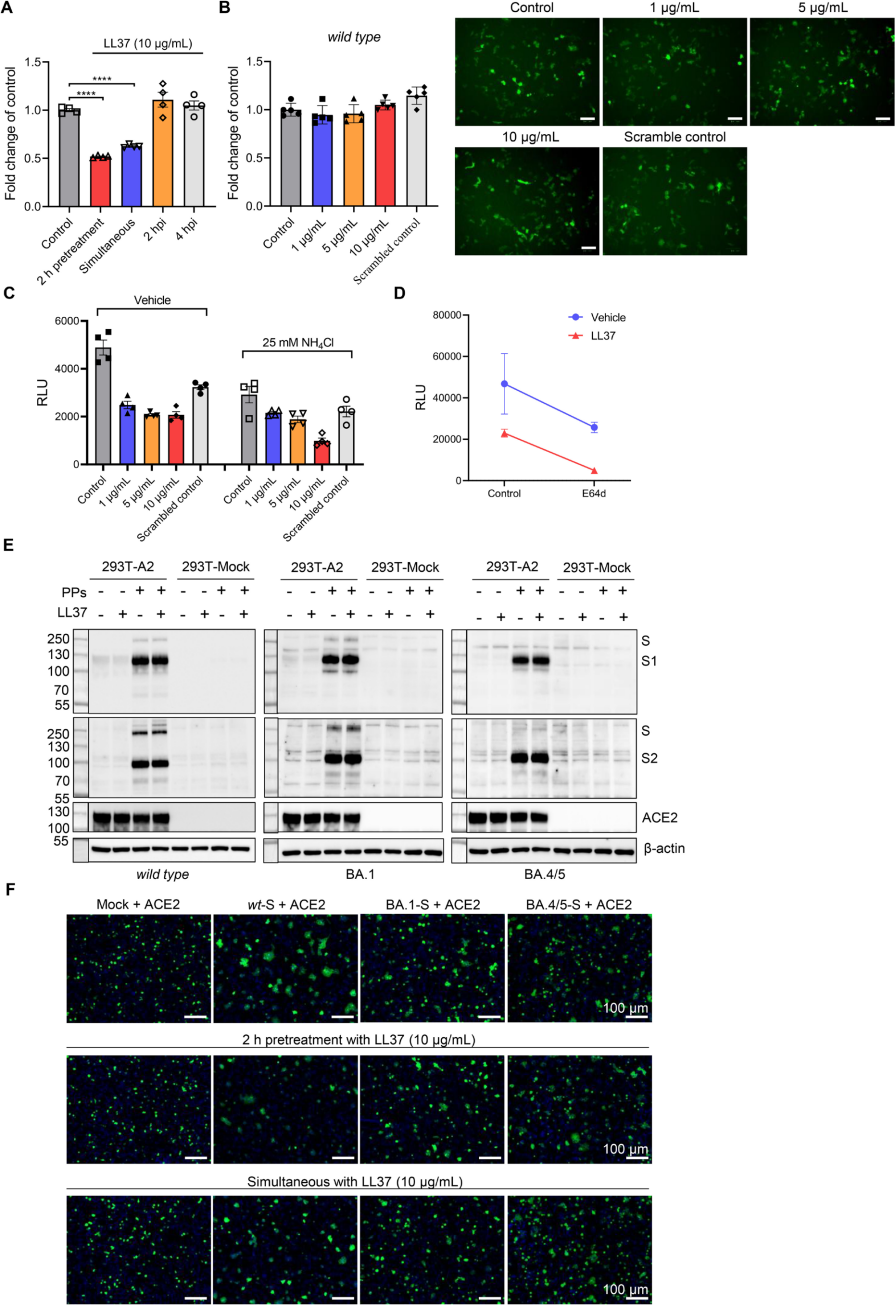
LL‐37 inhibited membrane fusion of SARS‐COV‐2 rather than Omicron variants that did not involve in the engagement with RBD domain or ACE2 receptor. (A) SARS‐CoV‐2 spike pseudoparticles were treated with LL‐37 (10 μg/mL) at indicated timepoints of 2 h pretreatment, simultaneous, 2 h post infection (hpi) or 4 hpi respectively. Infection rates were assessed at 2 days post infection by measuring the relative light unit (RLU) of luciferase activity. (B) 293T‐A2 cells were pretreated for 2 h with indicated concentrations of LL‐37, and SARS‐CoV‐2 spike pseudoparticles were then added. Infection rates were assessed at 2 days post infection by measuring the RLU of luciferase activity and fluorescent images were captured. KR‐37 was used as the scrambled control. (C‐D) LL‐37 inhibited the SARS‐CoV‐2 spike pseudoparticles entry not by the endosomal pathway. 293T‐A2 cells were pretreated for 1 h with 25 mM of NH_4_Cl (C), or for 2 h with 100 nM of E64d (D), and pseudoparticles pretreated for 2 h with indicated concentrations of LL‐37 were added. Infection rates were assessed at 2 days post infection by measuring the RLU of luciferase activity. (E) Western blotting analysis of S1 and S2 subunits levels of *wild type*, BA.1 and BA.4/5 pseudoparticles that attached to 293T‐A2 cells. The pseudoparticles were pretreated for 2 h with or without 10 μg/mL of LL‐37. 293T cells that transfected with a control vector (293T‐Mock) were used as a negative control. Three bands were captured as: S (∼250 kDa), S1 (∼110 kDa) and S2 (∼100 kDa). PPs: Pseudotyped particles. (F) Images of syncytia in the cell–cell fusion mediated by SARS‐CoV‐2 and Omicron spike. The effector cells were treated with LL‐37 (10 μg/mL) at indicated timepoints of 2 h pretreatment or simultaneous respectively. Data represent the mean ± SEM. Significance is indicated by ****p ≤ 0.0001.

The main mechanism of LL‐37 against SARS‐CoV‐2 is to block the interaction between the receptor binding domain (RBD) of spike S1 and the ACE2 receptor by binding to the RBD and/or ACE2 [36, 37, 41, 42]. To this end, we performed the time‐of‐addition experiment and revealed that LL‐37 prevented SARS‐CoV‐2 spike pseudovirus entry only if added prior (2 h) to or during infection but not if added 2 h post infection (hpi) and 4 hpi (Figure 2A); LL‐37 showed no inhibition of SARS‐CoV‐2 spike pseudovirus when only pretreated 293T cells that stably express human ACE2 (293T‐A2) (Figure 2B). In addition, we pretreated 293T‐A2 cells with NH_4_Cl or E64d (a cysteine protease inhibitor) respectively, to inhibit infection through the endosomal pathway (Figure 2C,D). The reduction of relative light unit (RLU) caused by LL‐37 showed no significant difference, suggesting that LL‐37 might perform the antiviral effect through the cell surface entry route that involves spike.

As mutations with alkaline amino acids (e.g., N440K, T478K, Q493R and Q498R) in RBD of Omicron spike elevate overall positive net charge [43], we hypothesised that in the present study, the potentially weakened ionic force between LL‐37 and RBD with those mutations might respond to the inability of LL‐37 against Omicron variants. Nevertheless, the attachment assay demonstrated that the plasma membrane‐attached spike (S1 and S2 subunits) of either SARS‐CoV‐2 or VOCs of Omicron BA.1 and BA.4/5 spike pseudoviruses pretreated with LL‐37 showed the equal quantity compared with that of vehicle control (Figure 2E). Next, we determined whether LL‐37 could perform on the membrane fusion process (Figures 2F and S3A,B). Cell–cell fusions mediated by spike of SARS‐CoV‐2 or Delta variant were inhibited by the addition of LL‐37, and pretreatment of spike‐expressing cells with LL‐37 exhibited stronger inhibition of fusion than treatment during infection, consistent with our results of the time‐of‐addition experiment. Expectedly, we verified the compromised inhibition of the BA.1‐ and BA.4/5‐spike induced cell–cell fusions by LL‐37, although the reduced background syncytia were observed in these two Omicron variants especially in BA.1 compared with that of SARS‐CoV‐2 or Delta variant (Figure 2F). These findings suggested that LL‐37 might inhibit the entry of SARS‐CoV‐2 and VOCs rather than Omicron variants, through blocking the membrane fusion process but not involving the engagement with the RBD domain or ACE2 receptor.

### 2.3 LL‐37 Blocks the TMPRSS2‐Mediated S2' Priming of SARS‐CoV‐2 S2 Subunit

**FIGURE 3:**
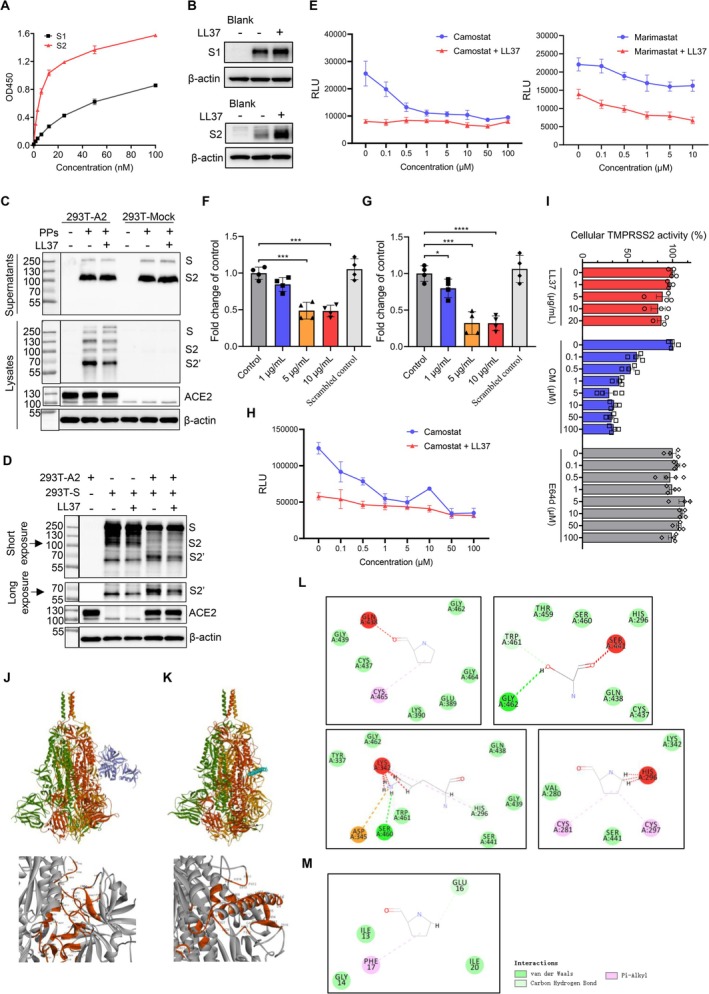
LL‐37 inhibited the TMPRSS2‐mediated S2' priming of SARS‐CoV‐2 S2 subunit. (A, B) LL‐37 exhibited much stronger binding to SARS‐CoV‐2 S2 subunit than S1 subunit. ELISA analysis of S1 and S2 subunits that bound to the immobilised LL‐37 (A) and western blotting analysis of S1 and S2 subunits levels that attached to 293T‐A2 cells facilitated by 10 μg/mL of LL‐37 (B). (C, D) LL‐37 inhibited the generation of S2' fragment of SARS‐CoV‐2 spike. Western blotting analysis of S2 subunit and S2' levels in the supernatant and 293T‐A2 cells which were co‐cultured with pseudoparticles (pretreated for 2 h with or without 10 μg/mL of LL‐37) for 8 h (C), or levels in the adherent syncytia of the spike‐expressing 293T cells (pretreated for 2 h with or without 20 μg/mL of LL‐37) and 293T‐A2 cells which were co‐cultured for 8 h (D). 293T cells that transfected with a control vector (293T‐Mock) were used as a negative control. Three bands were captured as: S (∼250 kDa), S2 (∼100 kDa) and S2' (∼68 kDa). PPs: Pseudotyped particles. (E) 293T‐A2 cells were pretreated for 2 h with indicated concentrations of camostat mesylate (CM) or marimastat, and then SARS‐CoV‐2 spike pseudoparticles pretreated for 2 h with 10 μg/mL of LL‐37 were added to cells. Infection rates were assessed at 2 days post infection by measuring the RLU of luciferase activity. (F‐G) SARS‐CoV‐2 spike pseudoparticles were pretreated for 2 h with indicated concentrations of LL‐37, and added to 293T‐A2T2 (F) or 293T‐A2 cells. Infection rates were assessed at 2 days post infection by measuring the RLU of luciferase activity. The RLU that was only induced by TMPRSS2 overexpression was calculated via subtracting of RLU in 293T‐A2T2 from that in 293T‐A2 cells (G). (H) 293T‐A2T2 cells were pretreated for 2 h with indicated concentrations of CM, and then SARS‐CoV‐2 spike pseudoparticles pretreated for 2 h with 10 μg/mL of LL‐37 were added to cells. Infection rates were assessed at 2 days post infection by measuring the RLU of luciferase activity. (I) TMPRSS2 activity measurement. LL‐37 showed no effect on the cellular TMPRSS2 activity of 293T‐T2 cells. (J‐K) Protein–protein docking analysis of TMPRSS2‐SARS‐CoV‐2 spike (J) and LL‐37‐SARS‐CoV‐2 spike (Pose 1, K). TMPRSS2 and LL‐37 were coloured as blue and cyan respectively. The binding surface was enlarged and highlighted as red, and the important binding residues were labelled. (L–M) Calculated 2D interactions of TMPRSS2 in complex with SARS‐CoV‐2 spike residues of Pro809, Ser810, Lys811 and Pro812 (L), and LL‐37 in complex with SARS‐CoV‐2 spike residues of Pro812 (M). Data represent the mean ± SEM. Significance is indicated by *p ≤ 0.05, ***p ≤ 0.001, ****p ≤ 0.0001.

To investigate how LL‐37 inhibits the SARS‐CoV‐2 spike‐induced membrane fusion, we performed a binding assay to characterise the interaction between the spike of SARS‐CoV‐2 and LL‐37. As shown in Figure 3A, the spike S2 subunit exhibited much stronger binding to immobilised LL‐37 in a dose‐dependent manner than S1 subunit. LL‐37 can attach to lipid bilayer via the C‐terminal VPRTES tail [44] and bridge between the SARS‐CoV‐2 spike and cell surface independent of the ACE2 expression (Figure S4A–C). By leveraging this feature, we further validated the S2 subunit rather than the S1 subunit that bound to the plasma membrane facilitated by LL‐37 (Figures 3B and S4B,C), in line with our binding assay analysis.

The unilateral change of the S2 subunit, *i.e*. S2' fragment which is processed mainly by TMPRSS2 of host cells, is of the utmost importance for the subsequent membrane fusion [4, 45, 46, 47, 48]. Therefore, in the next set of experiments, we explored whether LL‐37 could inhibit the S2' fragment generation. We collected these pseudovirus‐adherent cells and also adherent syncytia of the spike‐expressing 293T cells and 293T‐A2 cells respectively, and immunoblotted for cleaved spike species using a rabbit polyclonal antibody specifically detecting the S2 ectodomain (686 to 1208) as previously described [45]. Upon addition of pseudovirus or spike‐expressing 293T cells onto the 293T‐A2 cells but not 293T cells, the SARS‐CoV‐2 spike S2 subunit was cleaved to S2' (∼68 kDa) in a time‐dependent manner, and pretreatment with LL‐37 remarkably reduced the generation of S2' fragment (Figures 3C,D and S5A,B). The virions retained in the supernatant showed no cleavage in S2 subunit (Figures 3C and S5A). Moreover, the Delta variant pseudovirus performed the consistent result with that of SARS‐CoV‐2 (Figure S6). Since the spike was autocleaved or cleaved into S1 and S2 fragments when encountered with ACE2 receptor immediately (within 1 h) regardless of LL‐37 pretreatment, we excluded the effect of LL‐37 on the polybasic cleavage site between S1 and S2 boundary.

We next carried out inhibition experiments to identify which type of hydrolytic enzymes that LL‐37 may interfere with. We chose two types of commercially available inhibitors: (1) camostat mesylate (CM), a broad‐spectrum serine protease inhibitor. CM shows antiviral activity against SARS‐CoV‐2 through inhibiting TMPRSS2 and has been repositioned as a clinical candidate for treating COVID‐19 [4, 49]. (2) marimastat, a broad‐spectrum inhibitor of MMPs. MMPs are considered alternative hydrolases for S2' priming when the target cells express insufficient TMPRSS2 [50, 51]. Marimastat shows inhibitory activity against SARS‐CoV‐2‐induced membrane fusion at sub‐micromolar levels [50, 51]. As shown in Figure 3E, CM or marimastat (10 μM) reduced about 60% and 25% of SARS‐CoV‐2 spike pseudoviruses‐induced RLU respectively; however, 10 μg/mL of LL‐37 reduced the RLU that completely substituted the reduction caused by CM even though the concentration of CM reached 100 μM but not the marimastat, suggesting that LL‐37 might be involved in the interference of S2' priming mediated by TMPRSS2‐related enzymes. Because of low levels of TMPRSS2 expression in 293T and 293T‐A2 cells, we overexpressed TMPRSS2 in 293T‐A2 cells (293T‐A2T2, Figure S7A,B). In line with our entry inhibition analysis in 293T‐A2 cells, LL‐37 inhibited 52% of SARS‐CoV‐2 spike pseudovirus entry into 293T‐A2T2 cells (Figure 3F) and notably, inhibited about 70% of RLU which was only caused by the overexpression of TMPRSS2 (Figure 3G), together with the decrease of RLU by LL‐37 that almost substituted the reduction caused by CM (Figure 3H).

Next question is how LL‐37 inhibits the TMPRSS2‐mediated S2' priming. First, we demonstrated whether LL‐37 inhibits the proteolytic activity of TMPRSS2. The protease was overexpressed in 293T cells (293T‐T2, Figure S7B) and incubated with LL‐37, CM (as a positive control) or E64d (as a negative control). Enzymatic activity was assessed by adding a specific substrate that emits fluorescence after proteolytic cleavage. As shown in Figure 3I, CM but not LL‐37 or E64d suppressed cellular TMPRSS2 activity at sub‐micromolar concentrations, therefore we excluded the inhibition of TMPRSS2 activity by LL‐37. Then, we carried out molecular simulation to investigate the interaction between the SARS‐CoV‐2 spike and LL‐37 or TMPRSS2. Rigid‐body protein–protein docking was performed using the ZDOCK algorithm and optimal poses were selected from the largest cluster with a high ZRANK score. The conformation of the complex between TMRPSS2 and SARS‐CoV‐2 in selected docking pose revealed that the S2' site (Arg815/Ser816) was present at the flexible loops and interacted with the catalytic domain of TMPRSS2, including hydrogen bond, VDW (Van der Waals' force) and pi‐alkyl interactions with Pro809, Ser810, Lys811 and Pro812 of the SARS‐CoV‐2 spike (Figure 3J,L). LL‐37 in three selected docking poses (hereafter referred to as Pose 1, Pose 2 and Pose 3) could interact with Pro812 of the SARS‐CoV‐2 spike, leading to the formation of steric hindrance that might render TMRPSS2 unable to interact with S2' site (Figures 3K,M and S8A–D). These results demonstrated that LL‐37 might bind to SARS‐CoV‐2 spike S2 subunit and occupy the sites where TMPRSS2 binds to, leading to the inhibition of TMPRSS2‐mediated S2' priming process.

### 2.4 LL‐37 Shows Weak Inhibition of Membrane Fusion Induced by Omicron Variants

**FIGURE 4:**
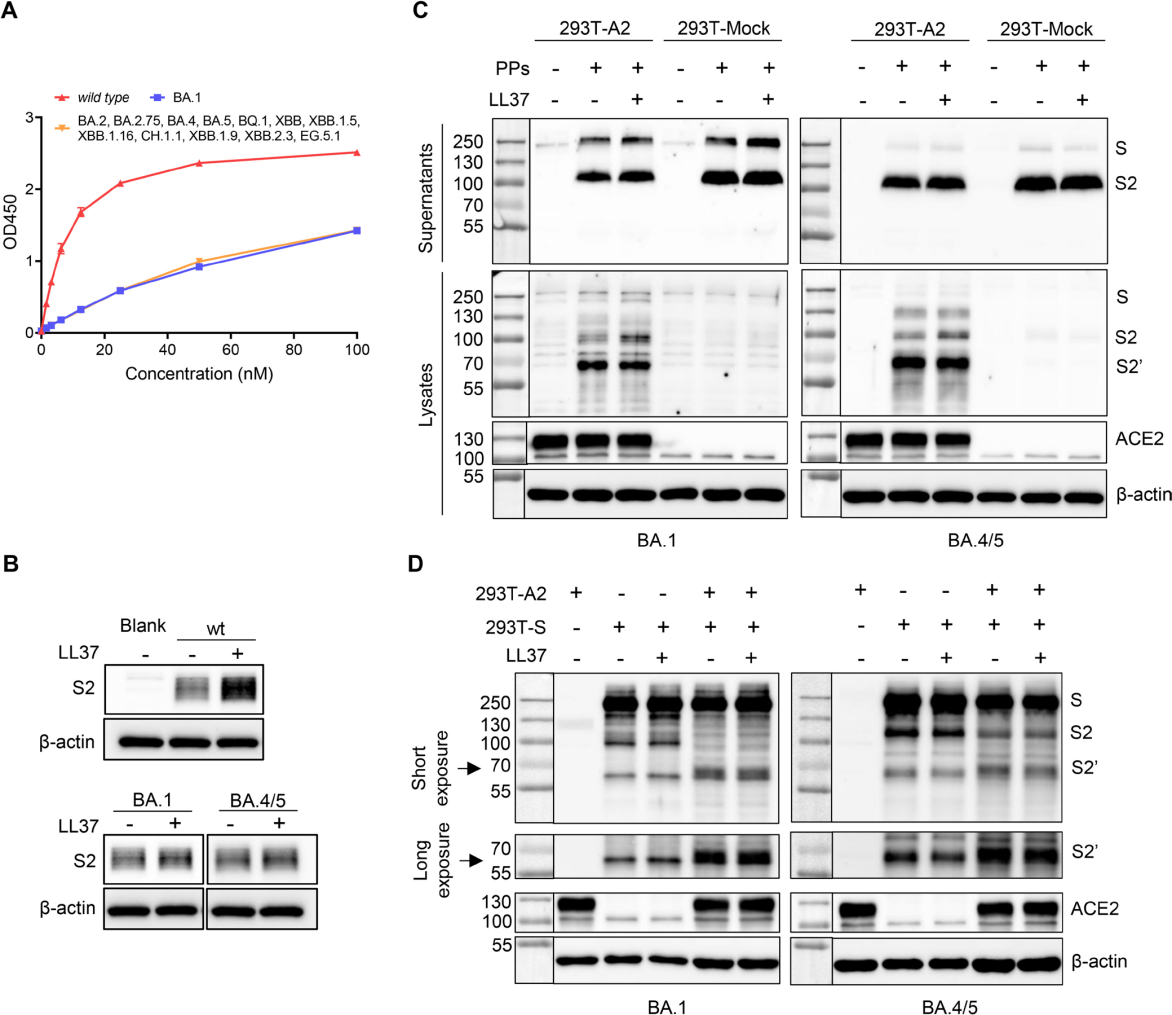
LL‐37 showed weak binding to S2 subunit of Omicron variants and did not inhibit its generation of S2' fragment. (A) ELISA analysis of indicated S2 subunits that bound to the immobilised LL‐37. (B) Western blotting analysis of indicated S2 subunits levels that attached to 293T‐A2 cells facilitated by 10 μg/mL of LL‐37. (C‐D) LL‐37 could not inhibit the generation of S2' fragment of BA.1 and BA.4/5 spike. Western blotting analysis of S2 subunit and S2' levels in the supernatant and 293T‐A2 cells which were co‐cultured with pseudoparticles (pretreated for 2 h with or without 10 μg/mL of LL‐37) for 8 h (C), or levels in the adherent syncytia of the spike‐expressing 293T cells (pretreated for 2 h with or without 20 μg/mL of LL‐37) and 293T‐A2 cells which were co‐cultured for 8 h (D). 293T cells that transfected with a control vector (293T‐Mock) were used as a negative control. Three bands were captured as: S (∼250 kDa), S2 (∼100 kDa) and S2' (∼68 kDa). PPs: Pseudotyped particles. Data represent the mean ± SEM.

Given that LL‐37 could not inhibit the entry of Omicron variants, we next investigated whether the TMPRSS2‐mediated S2' priming process of Omicron variants was unaffected by LL‐37. As shown in Figure 4A, the S2 subunits of spike of Omicron variants including BA.1, and BA.2, BA.2.75, BA.4, BA.5, BQ.1, XBB, XBB.1.5, XBB.1.16, CH.1.1, XBB.1.9, XBB.2.3 and EG.5.1 (sharing the identical amino acid sequence for S2 subunit), exhibited much lower binding to immobilised LL‐37 in a dose‐dependent manner than that of the SARS‐CoV‐2 (*wild type*). Also, the S2 subunit of SARS‐CoV‐2 rather than Omicron variants obviously bound to the plasma membrane with facilitation by LL‐37 (Figure 4B). Subsequently, we performed co‐incubation of pseudoviruses or 293T cells that express spike of Omicron variants (i.e., BA.1 or BA.4/5) and 293T‐A2 cells. As before, the S2 subunit was cleaved to S2' (∼68 kDa) in a time‐dependent manner, and the virions retained in the supernatant showed no cleavage in S2 subunit; however, pretreatment with LL‐37 did not significantly reduce the S2' fragment generation (Figures 4C,D and S9A–D).

### 2.5 N764K Mutation Within the S2 Subunit Is Essential for Omicron Variants to Diminish the LL‐37 Inhibitory Effect

**FIGURE 5:**
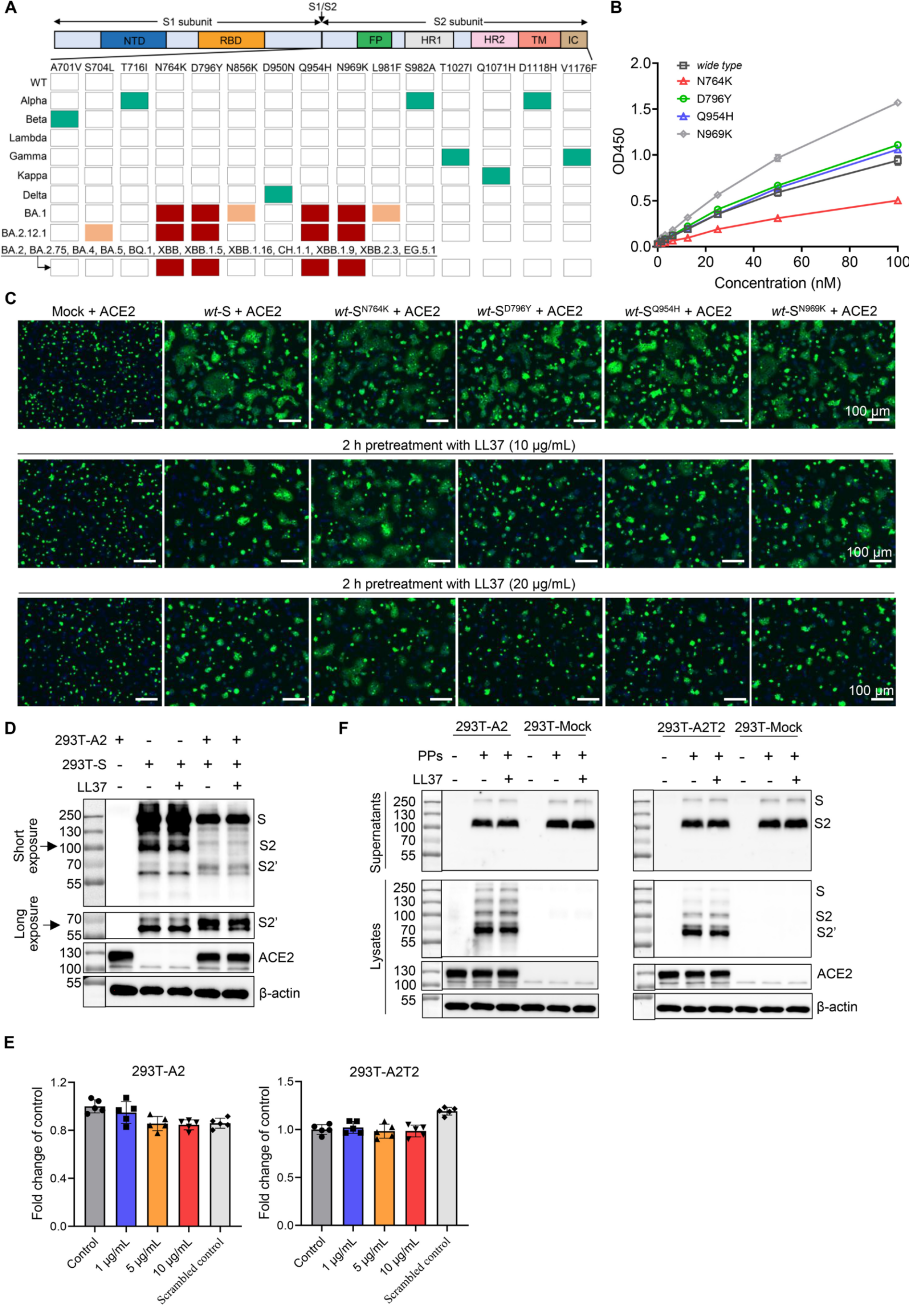
N764K mutation within the S2 subunit was essential for Omicron variants to diminish the LL‐37 inhibitory effect. (A) Image of mutations in S2 subunits of SARS‐CoV‐2 and VOCs. The mutations were indicated as colours: Dark cyan for Alpha, Beta, Lambda, Gamma, Kappa and Delta variants; dark red for the identical mutations of Omicron variants; orange for individual mutations of Omicron variants. (B) ELISA analysis of indicated S2 subunits that bound to the immobilised LL‐37. (C) Images of syncytia in the cell–cell fusion mediated by SARS‐CoV‐2 spike and mutated spikes. The effector cells were pretreated for 2 h with LL‐37 (10 or 20 μg/mL). (D) Western blotting analysis of S2 and S2' subunit levels in the adherent syncytia of the spike‐expressing 293T cells (with N764K mutation, pretreated for 2 h with or without 20 μg/mL of LL‐37) and 293T‐A2 cells which were co‐cultured for 8 h. Three bands were captured as: S (∼250 kDa), S2 (∼100 kDa) and S2' (∼68 kDa). (E) SRAS‐CoV‐2 spike pseudoparticles with N764K mutation were pretreated for 2 h with indicated concentrations of LL‐37, and then added to the 293T‐A2 or 293T‐A2T2 cells. Infection rates were assessed at 2 days post infection by measuring the RLU of luciferase activity. (F) Western blotting analysis of S2 subunit and S2' levels in the supernatant and 293T‐A2 or 293T‐A2T2 cells which were co‐cultured with N764K mutation of spike pseudoparticles (pretreated for 2 h with or without 10 μg/mL of LL‐37) for 8 h. 293T cells that transfected with a control vector (293T‐Mock) were used as a negative control. Three bands were captured as: S (∼250 kDa), S2 (∼100 kDa) and S2' (∼68 kDa). PPs: Pseudotyped particles. The BioRender.com. Data represent the mean ± SEM.

Next, we intended to see the effect of mutations within Omicron S2 subunit on LL‐37 inhibitory effect. The amino acid sequence alignment analysis indicated four mutations that only exist in Omicron and its subvariants, including N764K, D796Y, Q954H and N969K (Figure 5A). Of note, these four mutations also represent all mutations in S2 subunit of BA.2, BA.2.75, BA.4, BA.5, BQ.1, XBB, XBB.1.5, XBB.1.16, CH.1.1, XBB.1.9, XBB.2.3 and EG.5.1 variants. Therefore, we performed the Bac‐to‐Bac baculovirus expression system to obtain S2 proteins that possess indicated mutations respectively. As shown in Figure 5B, the S2 subunit with N764K mutation exhibited much lower binding to immobilised LL‐37 in a dose‐dependent manner than that of the SARS‐CoV‐2 S2 subunit (*wild type*); however, the S2 subunit with D796Y, Q954H or N969K mutation showed no difference or even enhanced binding to immobilised LL‐37 compared with that of the SARS‐CoV‐2 S2 subunit (*wild type*). Further, we determined the effect of LL‐37 on membrane fusion process (Figure 5C). In line with our binding analysis, pretreatment of spike‐expressing cells with LL‐37, including *wild type* spike, or spike with D796Y, Q954H or N969K mutation, exhibited significantly lower cell–cell fusion activity, but not the spike‐expressing cells with N764K mutation even the concentration of LL‐37 reached 20 μg/mL. Notably, mutations D796Y, Q954H and N969K, especially the N764K mutation, impaired the membrane fusion process compared with that of *wild type* to some extent. Subsequently, we performed co‐incubation of 293T cells that express spike with N764K, D796Y, Q954H, or N969K mutation and 293T‐A2 cells respectively, and collected these adherent syncytia. As shown in Figures 5D and S10A, the S2 subunit was cleaved to S2' (∼68 kDa), but pretreatment with LL‐37 did not significantly reduce the generation of S2' fragment in spike with N764K mutation. Interestingly, the spike with D796Y, Q954H, or N969K mutation showed less autocleaved S2 subunit as well as less S2' fragment generation when encountered ACE2 receptor, consistent with our observations of membrane fusion process.

To investigate the amino acid scanning mutagenesis, we performed a single mutation of N764K in spike and calculated the differences in folding and binding free energies between the *wild type* and the mutated structure of the complex respectively (Figure S10B,C). Three poses, *i.e*. Pose 1, Pose 2 and Pose 3, all showed increased folding free energy and destabilisation, leading to a decrease in the thermal stability of the complex. In addition, we also found that the N764K mutation in Pose 3 elevated the binding free energy, leading to a decrease in affinity and the weakening of interaction between spike and LL‐37. Finally, we constructed the luciferase‐ and EGFP‐expressing pseudoviruses of SARS‐CoV‐2 spike with the N764K mutation (SARS‐CoV‐2^N764K^). As shown in Figure 5E,F, LL‐37 did not significantly reduce the RLU induced by SARS‐CoV‐2^N764K^ as well as the generation of the S2' fragment when co‐incubated with 293T‐A2T2 or 293T‐A2 cells.

### 2.6 LL‐37 Enhances the Humoral Immune Response Induced by Spike of SARS‐CoV‐2 but Not the Omicron BA.4/5

An important issue is how Omicron variants progress to antibody evasion. To this end, we investigated whether LL‐37, an important part of innate immunity, could be involved in the adaptive immunity against SARS‐CoV‐2. We intramuscularly vaccinated mice with 5 μg of spike of SARS‐CoV‐2 or Omicron BA.4/5 formulated with LL‐37 following a prime‐boost regimen (one initial injection on day 0, and two boosters on day 14 and 21, respectively). As a control, the mice were injected with NS, LL‐37, or 5 μg of spike. Sera obtained on day 7 showed elevated IgM responses and titers to the SARS‐CoV‐2 spike, especially when formulated with the high concentration of LL‐37 (Figure S11A). The IgG responses to SARS‐CoV‐2 spike were gradually increased with peaking on day 14 when mice only received an initial injection (Figure S11B,C), and were enhanced by the high concentration of LL‐37 on day 21 when mice received one booster (Figure S11D); however, IgG responses and titers showed no significant difference compared with those of the spike control on day 28 when mice received two boosters (Figure S11E). Not surprisingly, compared with the control of BA.4/5 spike, the IgM and IgG responses and titers to the BA.4/5 spike showed no difference and low levels to background on day 7 or 14 when mice only received an initial injection (Figure S11F–H), and were even decreased when mice received one or two boosters (Figure S11I,J). Interestingly, the immunogenicity of only BA.4/5 spike was also significantly lower compared with that of SARS‐CoV‐2 spike. These results demonstrated that LL‐37 might be involved in the early adaptive immune response against SARS‐CoV‐2 but not the Omicron variants.

## 3 Discussion

In this study, we reported a novel mechanism for LL‐37 against SARS‐CoV‐2 and VOCs and how Omicron variants orientate themselves by extraordinary mutations towards the host immune defence. We found that LL‐37 predominantly bound to the S2 subunit of SARS‐CoV‐2 spike, occupying sites where TMPRSS2 typically binds and leading to the blocking of TMPRSS2‐mediated S2' priming and subsequent membrane fusion process; the mutation N764K within Omicron S2 subunit could reduce the LL‐37 binding and subsequent blocking of membrane fusion process. Importantly, we validated that the LL‐37 inhibition was not performed by interaction with the spike RBD domain or with host ACE2 that dominates the primary mechanism of LL‐37 against SARS‐CoV‐2 in recent studies [36, 37, 41, 42]. Moreover, LL‐37 enhanced early humoral immune responses against the SARS‐CoV‐2 spike protein in mice, but did not exhibit similar effects against Omicron BA.4/5 spike. These findings reveal a host regulatory mechanism underlying SARS‐CoV‐2 and VOCs infection and thus offer a promising strategy for the prevention and treatment of COVID‐19.

The coronavirus receptor ACE2 and the activator TMPRSS2 have been recognised as key molecules in SARS‐CoV‐2 infection. TMPRSS2 belongs to the type‐II transmembrane serine proteases (TTSPs) family, and proteolytically cleaves a conserved (R/K)‐(I/V) peptide, i.e., KR_815_‐S_816_F for spike S2 subunit, and is involved in priming virus entry and facilitating most respiratory transmissions of SARS‐CoV‐2 and the syncytia formation [48]. Amongst humans, high co‐expression levels of ACE2 and TMPRSS2 occur in the AT1 and AT2 pneumocytes, airway cells and nasal epithelial cells [23, 52, 53, 54]. Only basal activity levels of TMPRSS2 in host cells can support the SARS‐CoV‐2 [46, 47], influenza A [55, 56] and influenza B [56] entry and infection. Therefore, targeting TMPRSS2 may represent an alternative intervention to ameliorate SARS‐CoV‐2‐induced transmission and disease progression. For example, inhibitors peptidomimetics [57, 58], α1‐antitrypsin [59], homoharringtonine and halofuginone [60], against TMPRSS2 and related TTSPs exert high inhibition of SARS‐CoV‐2 entry and replication. Unlike to reducing the TMPRSS2 activity or expressing levels, we demonstrated that LL‐37 inhibited the TMPRSS2 catalytic process through binding to specific domains of spike and did not affect the TMPRSS2 activity, which protects the physiological function of the enzyme.

LL‐37 is mainly expressed by multiple immune cells like macrophages and neutrophils, and respiratory tract epithelial cells, and is important for the regulation of respiratory immunity and defence against bacteria and viruses [61]. Many factors, such as pro‐inflammatory cytokines and pathogen‐associated molecular patterns (PAMPs), can upregulate the LL‐37 expression, allowing for a high concentration at the site of infection [62]. LL‐37 performs pleiotropic effects on immunomodulation. For example, LL‐37 can bind to the DNA/RNA and augment signalling via TLR3, TLR7, or TLR9 that elevates the type I interferon response [63, 64], or directly bind with receptors such as FPRL‐1 and CXCR2 that are involved in the chemotaxis of monocytes, neutrophils and lymphocytes [65, 66]; otherwise, LL‐37 can also interact with the FPR‐2 receptor or prevent the translocation of NF‐kB p50 and p65 subunits, leading to immunosuppression [67, 68]. In the current study, we observed significantly decreased expression of LL‐37 in the respiratory tract of severe COVID‐19 patients. In addition to inhibiting TMPRSS2‐mediated S2' priming by LL‐37, we speculate that the basal levels of LL‐37, especially its levels in macrophages, might be vital for orchestrating the immune response to SARS‐CoV‐2 infection, and these patients with deficient levels of LL‐37 seem more prone to developing into a severe condition manifested as hyperinflammatory responses and immune dysfunction. Indeed, we saw that LL‐37 could enhance the early adaptive immune response against the SARS‐CoV‐2 spike. Further studies on the LL‐37‐mediated immunomodulation against SARS‐CoV‐2 are under investigation.

Nevertheless, we observed significantly decreased inhibition of Omicron variants by LL‐37. Many studies have demonstrated the mutations in Omicron for the increased spreading and robust immunoevasiveness [2, 43, 69]. In this study, we explored the underlying mechanism using point mutations in Omicron S2 subunit. We identified the mutation N764K as the most important for evading the LL‐37‐induced inhibition; however, we also found the decreased fusion when spike was mutated with D796Y, Q954H, or N969K, consistent with recent studies that show the reduced fusogenicity in Omicron variants especially in BA.1 [23, 70]. Indeed, the fusogenicity and replication are enhanced from BA.1 to BA.5 [71]. In addition, we found that LL‐37 could not enhance the adaptive immune response against Omicron spike. We speculate that the evolution of mutation profile with to adopt beneficial traits in Omicron variants can balance multiple matters, such as the fusogenicity, replication and evasion of host immune defences such as LL‐37 in the current study. However, several limitations remain to be addressed in the future, including (i) effects of mutation N764K on spike conformation and LL‐37 distribution; (ii) a detailed molecular understanding of the phenotypic impact of complex sets of mutations; (iii) identifying, enhancing, or modulating specific activity in LL‐37 by mutations that can inhibit most VOCs including Omicron variants.

In summary, this study discloses a previously unknown pathophysiological mechanism underlying the anti‐SARS‐CoV‐2 and VOCs activity by LL‐37 and how Omicron variants evade the inhibition by LL‐37. The findings may be of importance to understand the host response against TMPRSS2‐dependent viruses and highlight the potential clinical application for LL‐37 as an antiviral countermeasure.

## 4 Materials and Methods

### 4.1 Ethics and Biosafety Statement

All animal studies carried out were approved by the Animal Care and Use Committee of Sichuan University (Chengdu, Sichuan, China). Transgenic human ACE2 mice were approved by the Animal Care and Use Committee, the Institute of Laboratory Animal Science, Peking Union Medical College, China. All procedures involved in the live SARS‐CoV‐2 and VOCs were reviewed and approved by the Institutional Animal Care and Use Committee of the Institute of Medical Biology, Chinese Academy of Medical Science, and performed in the ABSL‐3 facility of Kunming National High‐level Biosafety Primate Research Center, Yunnan, China.

### 4.2 Peptides Synthesis

LL‐37 (sequence: LLGDFFRKSKEKIGKEFKRIVQRIKDFLRNLVPRTES) and a scrambled control peptide KR‐37 (sequence: KRKSFDGFKELILKGRQEIKFKRDIFLVNLRETRPSV) were synthesised by GL Biochem (China) and analysed by reversed phase high‐performance liquid chromatography (RP‐HPLC) and mass spectrometry to confirm their purity greater than 98%.

### 4.3 Cell Culture

HEK‐293T cells (CRL‐11268) and Sf9 cells (CRL‐1711) were purchased from American Type Culture Collection (ATCC). High Five cells (B85502) were purchased from ThermoFisher. 293T cells stably expressing human ACE2 (293T‐A2) were generated as previously reported [72]. 293T and 293T‐A2 cells were cultured in DMEM (Gibco, USA) and maintained in a humidified atmosphere containing 5% CO_2_ at 37°C. Sf9 and High Five cells were cultured in SIM SF medium and SIM HF medium respectively (Sino Biological, China) and maintained in a non‐humidified shaker at 28°C.

### 4.4 Plasmids and Transfection

SARS‐CoV‐2 spike encoding plasmids of pcDNA3.3‐CoV2‐D18 (addgene 170442), pcDNA3.3‐SARS2‐B.1.617.2 (addgene 172320) and pcDNA3.3‐SARS2‐Omicron‐BA.1 (addgene 180375) were gifts from David Nemazee [73, 74, 75], and the SARS‐CoV‐2 spike encoding plasmid of pCAGGS SARS‐CoV‐2 BA.4/5 (addgene 186031) was a gift from Marceline Côté [76]. The EGFP encoding plasmid (pEGFP‐C1) was a gift from the laboratory of Aiping Tong, Sichuan University. The human TMPRSS2 cDNA with HA tag was PCR amplified and cloned into the pcDNA3.1 vector as previously described [77]. Mutant vectors including SARS‐CoV‐2 spike with N764K, D796Y, Q954H and N969K were generated using a site‐directed gene mutagenesis kit (D0206S, Beyotime Biotechnology, China) according to the manufacturer's instruction. All constructs were confirmed by Sanger sequencing.

Transfections were performed using a PEI transfection reagent. Briefly, plasmids were mixed with PEI in a 3:1 ratio in Opti‐MEM I reduced serum medium (ThermoFisher, USA), incubated for 15 min at RT, and then added to cells dropwise. The transiently transfected cells were harvested for further experiments after 24 h of transfection.

### 4.5 Infection by Spike Pseudoviruses of SARS‐CoV‐2 and VOCs

Luciferase‐ and EGFP‐expressing spike pseudoviruses including SARS‐CoV‐2 and VOCs and the single N764K mutation of pseudovirus were purchased from Genomeditech (China). Briefly, the pseudovirus was pretreated for 2 h at 37°C with LL‐37 (0, 1, 5, 10 μg/mL, prepared with DMEM free of FBS), and then added to 293T‐A2 or 293T‐A2T2 cells (1.2 × 10^4^/well) in a black 96‐well plate for 48 h of incubation to express the luciferase. Finally, 100 μL of lysis reagent with luciferase substrate from a luciferase kit (Beyotime Biotechnology, China) was added, and the relative light unit (RLU) was determined by a multi‐mode microplate reader (PerkinElmer, USA). KR‐37 was used as a scrambled control. In one experiment, 293T‐A2 cells (1.2 × 10^4^/well) in a black 96‐well plate were pretreated for 2 h at 37°C with LL‐37 (0, 1, 5, 10 μg/mL, prepared with DMEM free of FBS), and then pseudovirus was added and incubated for 48 h as previously described.

For the time‐of‐addition experiment, groups were performed as follows: (1) pseudovirus was pretreated for 2 h at 37°C with 10 μg/mL of LL‐37 (prepared with DMEM free of FBS), and then added to 293T‐A2 cells; (2) pseudovirus and 10 μg/mL of LL‐37 (prepared with DMEM free of FBS) were added to 293T‐A2 cells simultaneously; (3) pseudovirus (prepared with DMEM free of FBS) was added to 293T‐A2 cells, and 10 μg/mL of LL‐37 was added 2 h post infection; (4) pseudovirus (prepared with DMEM free of FBS) was added to 293T‐A2 cells, and 10 μg/mL of LL‐37 was added 4 h post infection.

To study the involvement of LL‐37 in virus entry through the endosomal route, 293T‐A2 cells were pretreated for 1 h at 37°C with 25 mM of NH4Cl, or for 2 h at 37°C with 100 nM of E64d, and then pseudovirus (pretreated for 2 h at 37°C with 0, 1, 5, 10 μg/mL of LL‐37, prepared with DMEM free of FBS) was added to and incubated with cells for 48 h as previously described.

To study the involvement of LL‐37 in virus entry through the cell surface, 293T‐A2 or 293T‐A2T2 cells were pretreated for 2 h at 37°C with camostat mesylate (0, 0.1, 0.5, 1, 5, 10, 50, 100 μM, MedChemExpress) or marimastat (0, 0.1, 0.5, 1, 5, 10, 20 μM, MedChemExpress), and then pseudovirus (pretreated for 2 h at 37°C with 0, 1, 5, 10 μg/mL of LL‐37, prepared with DMEM free of FBS) was added to and incubated with cells for 48 h as previously described.

### 4.6 SARS‐CoV‐2 and VOCs Infection in Mice

Transgenic human ACE2 mice with a C57BL/6 background were intranasally instilled with original SARS‐CoV‐2 and VOCs of Delta and Omicron BA.1 respectively (5 × 10^4^ PFU in 40 μL of PBS per mouse). The vehicle control received an equal volume of PBS. Mice were euthanized on 3 dpi for lung processing.

### 4.7 Analysis of Single‐Cell RNA‐Seq Datasets

For bronchoalveolar lavage fluid (BALF), single‐cell RNA‐seq data were retrieved from published resources, including BALF from 6 severe and 3 moderate COVID‐19 patients and 3 healthy donors [78], and downloaded from Gene Expression Omnibus under the accession number GSE145926. Data and distribution of expression for *CAMP* gene process were performed by R.

### 4.8 Virus Attachment Assay

293T‐A2 cells (6 × 10^5^/well) in a 6‐well plate were infected with pseudovirus (pretreated for 2 h at 37°C with or without 10 μg/mL of LL‐37, prepared with DMEM free of FBS), and incubated for 1 h at 4°C to allow virus attachment. Then, cells were washed three times with ice‐cold PBS to remove the unbound virions. Cellular total protein was extracted, and the levels of bound virions were measured by western blotting using the antibodies specifically detecting the SARS‐CoV‐2 spike S1 and S2 subunits respectively.

### 4.9 Cell–Cell Fusion Assay

293T‐A2 cells were used as target cells, and 293T‐EGFP/S cells were used as effector cells. Briefly, 293T‐EGFP/S cells (2 × 10^4^/well) in a 96‐well plate were pretreated for 2 h at 37°C with or simultaneously treated with 10 or 20 μg/mL of LL‐37 (prepared with DMEM free of FBS), and then 293T‐A2 cells (2 × 10^4^/well) were added to co‐culture with effector cells for 4 or 6 h at 37°C. Subsequently, cells were stained for 10 min at 37°C with Hoechst (Beyotime biotechnology, China), and the syncytia formation was captured by an inverted fluorescence microscope (Olympus Corporation, Japan). 293T‐EGFP cells co‐cultured with target cells were used as a negative control.

### 4.10 S2' Fragment Measurement

SARS‐CoV‐2 and VOCs pseudoparticles including the original, Delta, BA.1 and BA.4/5, were pretreated for 2 h at 37°C with or without 10 μg/mL of LL‐37 (a total volume of 500 μL, prepared with FBS‐free DMEM), and then added to 293T‐A2 or 293T‐mock cells (6 × 10^5^/well) in a 6‐well plate to a final volume of 1 mL and incubated for 2 h, 4 h and 8 h at 37°C. The supernatant and 293T‐A2 cells were collected respectively for S2' fragment measurement by western blotting using the antibody specifically detecting the SARS‐CoV‐2 spike S2 subunit. Untreated 293T‐A2 or 293T‐mock cells were used as the blank control.

In addition, the transiently spike‐expressing (the original, BA.1 and BA.4/5) 293T cells (6 × 10^5^) were pretreated for 2 h at 37°C with or without 10 μg/mL of LL‐37 (a total volume of 500 μL, prepared with FBS‐free DMEM), and then added to 293T‐A2 cells (6 × 10^5^/well) in a 6‐well plate to a final volume of 1 mL and incubated for 2 h, 4 h and 8 h at 37°C. The cells were collected for S2' fragment measurement by western blotting using the antibody specifically detecting the SARS‐CoV‐2 spike S2 subunit. Untreated 293T‐A2 cells were used as the blank control.

### 4.11 Expression, Purification and Biotinylation of SARS‐CoV‐2 Spike S2 Proteins

SARS‐CoV‐2 original strain S2 (residues 686–1213 in spike protein, GenBank accession number: QHD43416.1), S2‐N764K (residues 686–1213 in spike protein and bearing N764K mutation), S2‐D796Y (residues 686–1213 in spike protein and bearing D796Y mutation), S2‐Q954H (residues 686–1213 in spike protein and bearing Q954H mutation) and S2‐N969K (residues 686–1213 in spike protein and bearing N969K mutation) proteins were expressed in High Five cells using the Bac‐to‐Bac baculovirus expression system (Invitrogen). Briefly, the coding sequence was cloned into a customised pFastBac1 vector and fused with an N‐terminal gp67 signal peptide that helps protein secretion. C‐terminal 6 × His and Avi tags were added for purification and biotinylation. Baculoviruses were produced by transfection of recombinant bacmids DNA into Sf9 cells and then were used to infect High Five cells. The supernatants of the infected High Five cells were harvested 2days post infection (dpi) at 27°C with 110 rpm of shake.

The S2 proteins were purified orderly by Ni‐TED agarose resin (NUPTEC, China), Superdex 200 Increase 10/300 GL column (GE Healthcare, USA) and HiTrap Q HP column (GE Healthcare, USA). Finally, the proteins were equilibrated in a buffer consisting of 20 mM Tris–HCl (pH 8.0) and 150 mM NaCl for further use. The purity of the proteins was measured by SDS–PAGE and visualised by staining with FastBlue (Biosharp, China) and by western blotting (Figure S12A).

The biotinylation of S2 proteins was performed using an Avi tag protein biotin labelling kit (Beyotime Biotechnology, China). Briefly, 20 μM of purified S2 protein was added to the reaction system and then incubated at 30°C for 1 h, followed by stopping the reactions with a 4°C treatment. The supernumerary biotin was removed by dialysis for 16 h at 4°C. The biotin labelling efficiency was detected by western blotting using the HRP‐labelled streptavidin (A0303, 1:2500, Beyotime Biotechnology, China, Figure S12B).

### 4.12 Binding of the S1 or S2 Subunit Proteins to LL‐37

To investigate the proteins of S1, S2 and S2 subunit with indicated mutations that bind to LL‐37, LL‐37 was performed to coat flat‐bottom 96‐well plates (ThermoFisher, USA) at a final concentration of 1 μg/mL in 50 mM carbonate coating buffer (pH 9.6) at 4°C overnight. Then blocking solution containing 1% BSA in PBST was added for 1 h of incubation at RT. Serially diluted biotinylated S1 and S2 proteins (Sino Biological, China), and S2 proteins with indicated mutations, were added and incubated for 1.5 h at 37°C. After three washes with PBST, streptavidin‐conjugated horseradish peroxidase (HRP) was added for 1 h of incubation at RT. Development was performed using 3,3′,5,5′‐tetramethyl biphenyl diamine (TMB) for 5 min of incubation, followed by the stop of reactions by 100 μL/well of stop solution (P0215, Beyotime Biotechnology, China). Absorbance was measured at 450 nm using a microplate reader (Biotek, USA).

In addition, we also performed western blotting analysis to measure the S1 or S2 subunit of SARS‐CoV‐2 and Omicron variants that attach to cell membranes bridged by LL‐37. Briefly, 1 μg of S1 or S2 protein (ACRO Biosystems, China) was incubated for 2 h at 37°C with 10 μg/mL of LL‐37 (a total volume of 500 μL, prepared with FBS‐free DMEM). Then the mixture was added to 293T‐A2 or 293T‐mock cells (6 × 10^5^/well) in a 6‐well plate to a final volume of 1 mL (prepared with FBS‐free DMEM), and incubated for 1 h at 4°C. The cells were washed three times with ice‐cold PBS and cellular total protein was harvested. The levels of attached S1 or S2 protein were measured by western blotting using the antibodies specifically detecting the SARS‐CoV‐2 spike S1 and S2 subunits respectively.

### 4.13 TMPRSS2 Activity Measurement

293T‐T2 cells (5 × 10^4^/well) in a black 96‐well flat‐bottom plate were treated for 2 h at 37°C with LL‐37 (0, 1, 5, 10, 20 μg/mL), camostat mesylate (0, 0.1, 0.5, 1, 5, 10, 50, 100 μM, MedChemExpress), or E64d (0, 0.1, 0.5, 1, 5, 10, 50, 100 μM, MedChemExpress). The peptide and inhibitors were prepared with DMEM free of FBS. To measure the proteolytic activity of TMPRSS2, the medium was replaced with 100 μL of DMEM free of FBS and phenol red and containing 100 μM of Boc‐Phe‐Ser‐Arg‐MCA (Peptide, Japan). The fluorescence intensity was measured after 2 h of incubation at 37°C using a microplate reader (Biotek, USA). The excitation/emission wavelengths were 360/460 nm. The values were expressed as a percent of fluorescence intensity relative to the control. Camostat mesylate and E64d were used as the positive and negative controls respectively. All procedures were carried out in the dark.

### 4.14 Spike Vaccine Formulation, Vaccinations of Mice and Antibody Titer Measurement

Spike recombinant proteins of original SARS‐CoV‐2 (40589‐V08B1) and Omicron of BA.4/5 (40589‐V08H32) were purchased from Sino Biological with a purity of more than 90%. The recombinant protein vaccine was prepared by mixing spike with LL37 at the mass ratio of 1:1, 1:2 or 1:8 respectively, and maintained for 15 min at RT to form spike‐LL‐37 complexes. We used 6–8‐week‐old female BALB/c mice for immunisation. The mice received immunisation by intramuscular injection of proteins of spike (5 μg per mouse), spike‐LL‐37 (normalised to the spike protein level in complexes, i.e., 5 μg of spike protein per mouse), or LL‐37 (normalised to the highest dose of LL‐37 in spike‐LL‐37 complexes, i.e., 40 μg of LL‐37 per mouse) on days 0, 14 and 21. PBS was used as a vehicle control. Blood samples were collected on days 7, 14, 21 and 28 via eye socket vein and centrifuged at 6000 rpm for 15 min for sera collection.

For IgG and IgM antibody titers measurement, spike recombinant protein of SARS‐CoV‐2 or Omicron BA.4/5 was performed to coat flat‐bottom 96‐well plates (ThermoFisher, USA) at a final concentration of 1 μg/mL in 50 mM carbonate coating buffer (pH 9.6) at 4°C overnight. Then blocking solution containing 1% BSA in PBST was added for 1 h of incubation at RT. Serially diluted serum was added and incubated for 1 h at 37°C. Antibodies, including goat anti‐mouse IgG and IgM HRP‐conjugated antibodies, were diluted 1:10,000 in blocking solution and added to wells (100 μL/well) for 1 h of incubation at 37°C. Development was performed using TMB for 10 min of incubation, followed by the stopping of reactions with 100 μL/well of stop solution (P0215, Beyotime Biotechnology, China). Absorbance was measured at 450 nm using a microplate reader (Biotek, USA).

### 4.15 Quantitative PCR (qPCR) and Western Blotting

Total RNA was isolated from mouse lung tissues using a Trizol method (ThermoFisher, USA) according to the manufacturer's instructions. First‐strand cDNA was synthesised using a PrimeScriptTM RT reagent kit (Takara, Japan). qPCR was performed in triplicate using SsoFast EvaGreen Supermix (Bio‐Rad, USA) at a 20 μL total volume on a Bio‐Rad iCycler RT–PCR detection system. Primers used in this study for *mus musculus* were as follows: *Camp* forward, 5′‐GCTGTGGCGGTCACTATCAC‐3′ and reverse, 5′‐TGTCTAGGGACTGCTGGTTGA‐3′; *β‐actin* forward, 5′‐GGCTGTATTCCCCTCCATCG‐3′ and reverse, 5′‐CCAGGTAACAATGCCATGT‐3′. The expression values of each replicate were normalised to *β‐actin* cDNA using the 2^−ΔΔCT^ method.

Total protein was extracted using the ice‐cold enhanced RIPA lysis buffer supplemented with a protease inhibitor cocktail (HY‐K0010, MedChemExpress, USA) and measured using the BCA method. Western blotting was performed using standard protocols according to the manufacturer's recommendations. Restore PLUS Western Blot Stripping Buffer (46430, ThermoFisher, USA) was used to strip the antibodies before re‐incubation of membranes. Primary antibodies used for western blot analysis included rabbit anti‐SARS‐CoV‐2 (2019‐nCoV) spike S1 (Sino Biological, 40591‐T62), rabbit anti‐SARS‐CoV‐2 (2019‐nCoV) spike S2 (Sino Biological, 40590‐T62), rabbit anti‐Cathelicidin/CLP (Abcam, ab207758), rabbit anti‐ACE2 (Sino Biological, 10108‐R003), rabbit anti‐TMPRSS2 (Abways, CY8435) and mouse anti‐β‐actin (HuaBio, HA601082).

### 4.16 Molecular Modelling of SARS‐CoV‐2 S Protein, LL‐37 and TMPRSS2

All computational studies were carried out using Discovery Studio (DS; Accelrys, San Diego, CA). The rigid‐body protein–protein docking algorithm ZDOCK was performed to systematically search the rotational and translational space of SARS‐CoV‐2 spike and TMPRSS2 or LL‐37. The initial structures were prepared based on X‐ray or cryo‐EM crystal structures: TMPRSS2 (PDB ID 7MEQ) and SARS‐CoV‐2 spike (PDB ID 6VSB). The unresolved residues were constructed by using the homology modelling module Modeller within DS. LL‐37 was constructed by pGenThreader (http://bioinf.cs.ucl.ac.uk/psipred/).

The spike was used as the receptor protein, and the TMPRSS2 or LL‐37 was used as the ligand protein. In dynamic simulations, pressure, temperature, constant molecular number and periodic boundary conditions were used. The CHARMM force field was employed for the protein. The energies of docked poses were calculated and filtered based on the ZDOCK score. In addition, we performed the Calculate Mutation Energy protocol to evaluate the effect of the N764K mutation on the binding affinity and folding energy of molecular partners in protein–protein and protein‐ligand complexes. In brief, combinatorial amino‐acid scanning mutagenesis on a set of selected amino‐acid residues was used by mutating them to one or more specified amino‐acid types. The energy effect of the N764K mutation on the binding affinity and folding energy (mutation energy, ΔΔGmut) was calculated as the difference between the binding free energy and folding free energy in the mutated structure and *wild‐type* protein respectively. More specific parameters and processes were prepared according to the previous description [79].

### 4.17 Statistical Analysis

All data were analysed using one‐way ANOVA and Student's unpaired *t*‐test (GraphPad InStat Software Inc., CA, USA). Results are presented as the means ± SEM. *p* < 0.05 was considered significant (significance is denoted as follows: *p ≤ 0.05; **p ≤ 0.01; ***p ≤ 0.001; ****p ≤ 0.0001).

## Author Contributions

**Zhenfei Bi and Xiawei Wei:** conceptualization. **Zhenfei Bi, Wenyan Ren, Guangwen Lu and Yuquan Wei:** methodology. **Zhenfei Bi, Wenyan Ren, Hao Zeng, Yuanyuan Zhou, Jian Liu, Zimin Chen, Xindan Zhang and Xuemei He:** investigation. **Zhenfei Bi, Wenyan Ren, Hao Zeng, and Yuanyuan Zhou:** visualisation. **Zhenfei Bi and Xiawei Wei:** funding acquisition. **Xiawei Wei:** project administration. **Xiawei Wei:** supervision. **Zhenfei Bi:** writing – original draft. **Zhenfei Bi, Wenyan Ren, and Xiawei Wei:** writing – review and editing.

## Conflicts of Interest

The authors declare no conflicts of interest.

## Supporting information


**Data S1.** Supporting Information.

## Data Availability

The data that support the findings of this study are available from the corresponding author upon reasonable request.
