# Supplementary material for: LL‐37 Inhibits TMPRSS2‐Mediated S2' Site Cleavage and SARS‐CoV‐2 Infection but Not Omicron Variants
Source: Cell Prolif. 2025 May 15;58(12):e70060. doi: 10.1111/cpr.70060 (PMC12686126; doi:10.1111/cpr.70060)
Supplement: Supplementary file 1 — Data S1. Supporting Information. [file CPR-58-e70060-s001.pdf]

Supplementary Materials for

LL-37 inhibits TMPRSS2-mediated S2' site cleavage and SARS-CoV-2 infection but not Omicron variants

Zhenfei Bi, Wenyan Ren, Hao Zeng, Yuanyuan Zhou, Jian Liu, Zimin Chen, Xindan Zhang, Xuemei He, Guangwen Lu, Yuquan Wei, Xiawei Wei

Corresponding author: Xiawei Wei

Email: xiaweiwei@scu.edu.cn

This PDF file includes:

Figures S1 to S12.

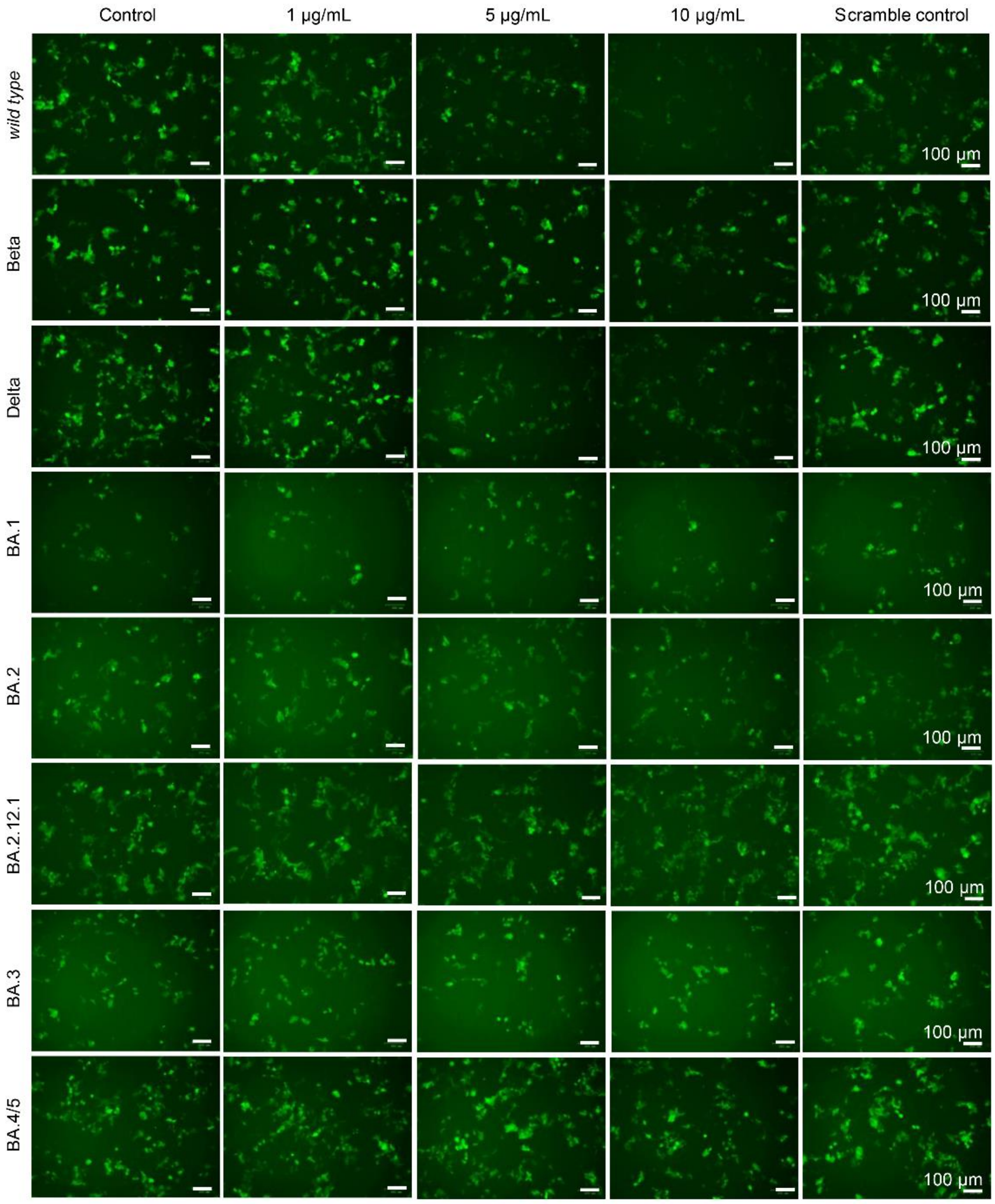

Figure S1. LL-37 inhibited the entry of SARS-CoV-2 spike pseudoparticles and VOCs but not Omicron variants. SRAS-CoV-2 and VOCs spike pseudoparticles were pretreated for 2 h with indicated concentrations of LL-37, and then added to the 293T-A2 cells. Fluorescent images were captured at 2 days post infection.

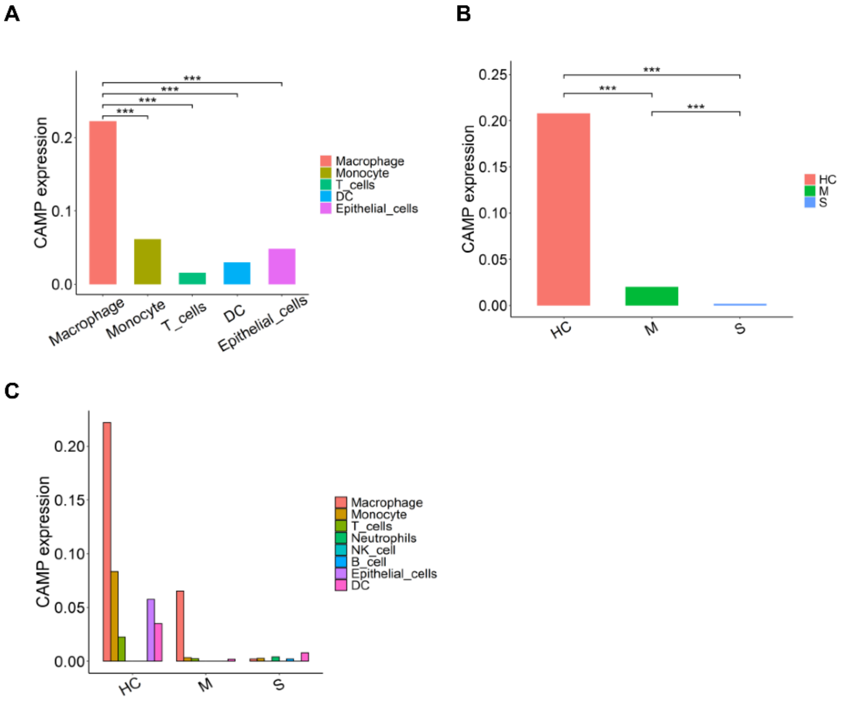

Figure S2. ***CAMP*** **expression levels by single-cell RNAseq.** (**A-C**) Long-normalized expression of *CAMP* gene in single-cell RNAseq data for indicated cell types of normal BALF (**A**), for multiple conditions of BALF (healthy, moderate and severer) (**B**) and for indicated cell types of multiple conditions of BALF (healthy, moderate and severer) (**C**). HC: healthy control; M: moderate COVID-19 patients; S: severe COVID-19 patients.

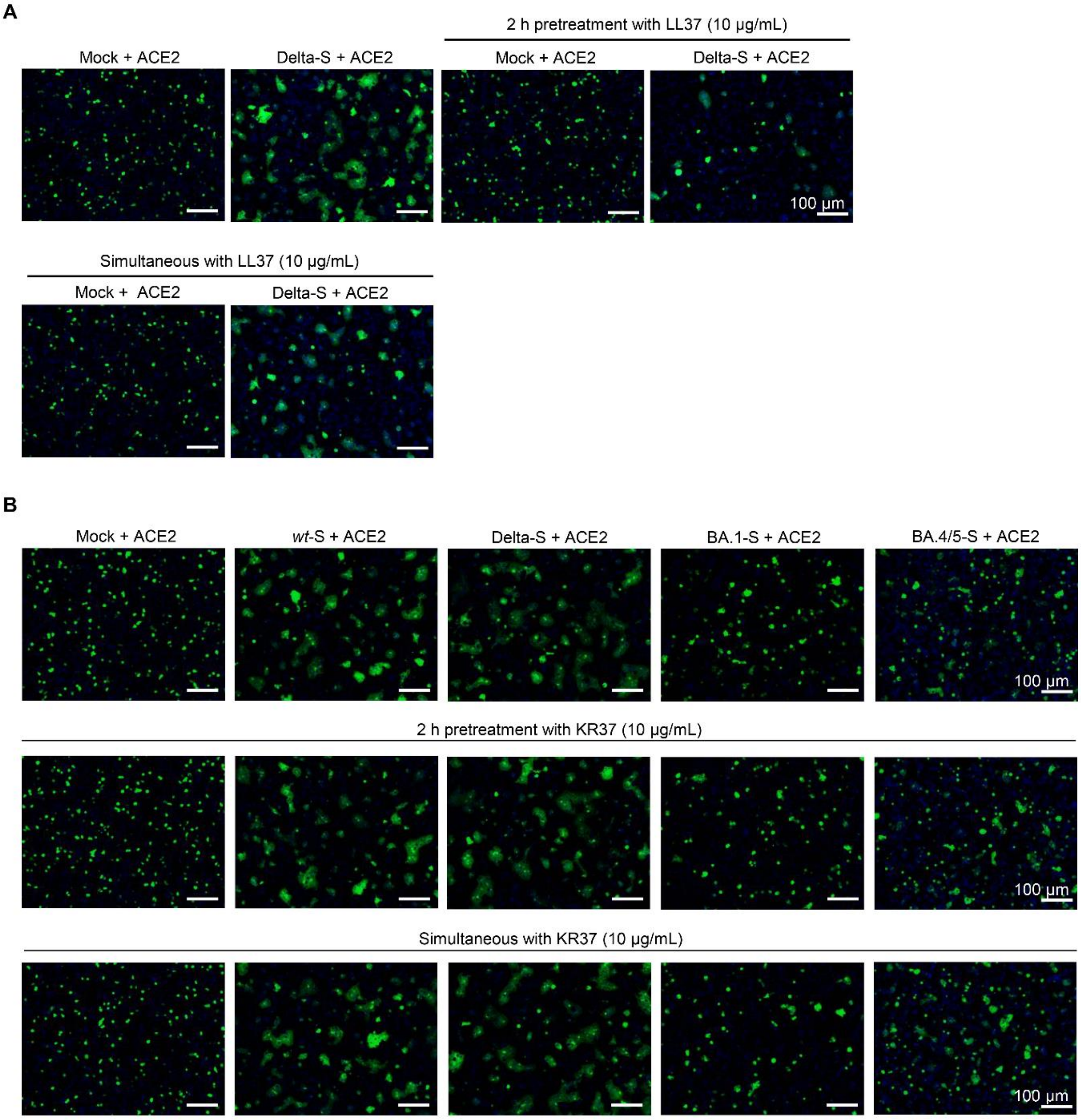

Figure S3. LL-37 inhibited the cell-cell fusion mediated by Delta spike protein and KR-37 showed no inhibition to cell-cell fusion mediated by spike protein of SARS-CoV-2, Delta or Omicron variants. (**A-B**) Images of syncytia in the cell-cell fusion mediated by spike protein of SARS-CoV-2, Delta, or Omicron BA.1 or BA.4/5. The effector cells were treated with 10 μg/mL of LL-37 (**A**) or 10 μg/mL of KR-37 (**B**) at indicated timepoints of 2 h pretreatment or simultaneous respectively. 293T cells that transfected with a control vector (293T-Mock) were used as a negative control.

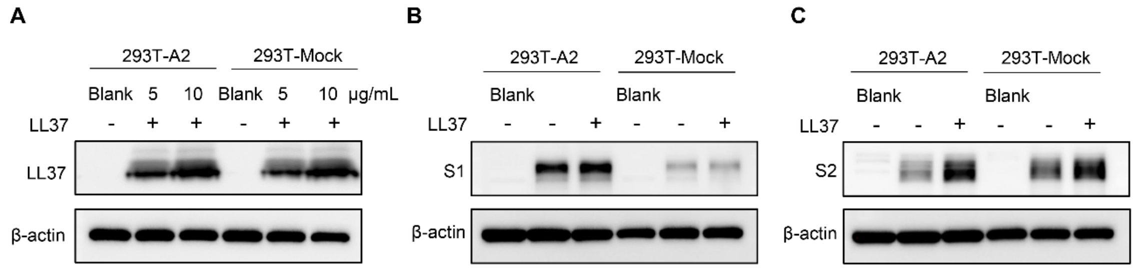

Figure S4. LL-37 could attach to the cell membrane independent of the ACE2 expression and bridge between the S2 subunit of SARS-CoV-2 and cell surface. (**A**) Western blotting analysis of LL-37 levels that attached to cell membranes. 293T-A2 cells were treated for 2 h with indicated concentrations of LL-37 and were collected for immunoblotting. 293T-Mock cells were used as the control. (**B**-**C**) Western blotting analysis of S1 (**B**) and S2 (**C**) subunits levels that attached to 293T-A2 or 293T-Mock cells facilitated by 10 μg/mL of LL-37. LL-37 bridged more S2 subunits than S1 subunits that bound to the plasma membranes of both 293T-A2 and 293T-Mock cells.

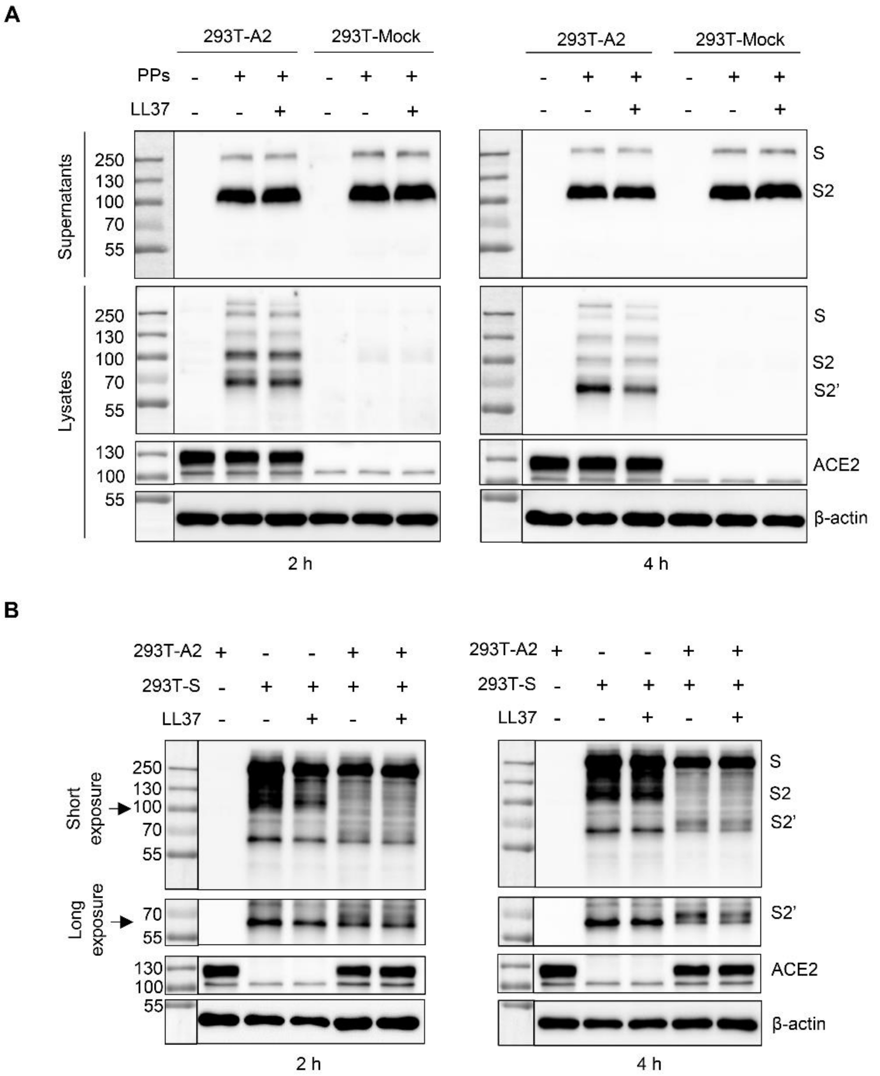

Figure S5. LL-37 inhibited the generation of S2' fragment of SARS-CoV-2 spike protein. (**A-B**) Western blotting analysis of S2 subunit and S2' levels in the supernatant and 293T-A2 cells which were co-cultured with pseudoparticles (pretreated for 2 h with or without 10 µg/mL of LL-37) for 2 h and 4 h (**A**), or levels in the adherent syncytia of the spike-expressing 293T cells (pretreated for 2 h with or without 20 µg/mL of LL-37) and 293T-A2 cells which were co-cultured for 2 h and 4 h (**B**). 293T cells that transfected with a control vector (293T-Mock) were used as a negative control. Three bands were captured as: S (~250 kDa), S2 (~100 kDa), and S2' (~68 kDa). PPs: pseudotyped particles.

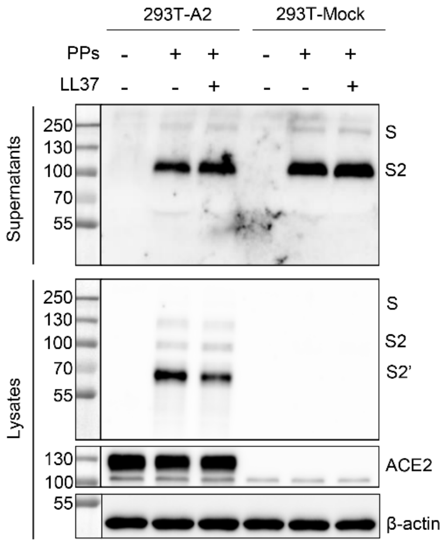

Figure S6. LL-37 inhibited the generation of S2' fragment of Delta variant spike protein. Western blotting analysis of S2 subunit levels in the supernatant and 293T-A2 cells which were co-cultured with Delta spike pseudoparticles (pretreated for 2 h with or without 10 μg/mL of LL-37) for 8 h. 293T cells that transfected with a control vector (293T-Mock) were used as a negative control. Three bands were captured as: S (~250 kDa), S2 (~100 kDa), and S2' (~68 kDa). PPs: pseudotyped particles.

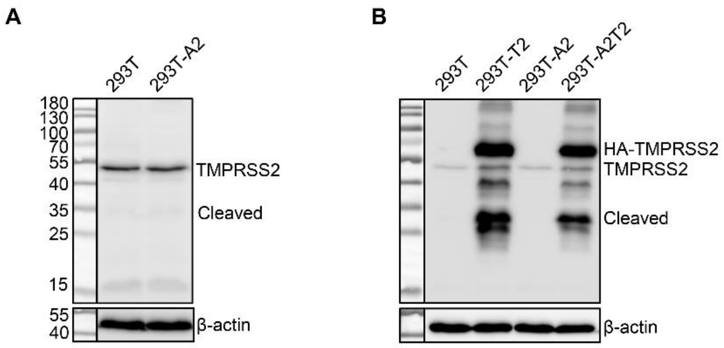

Figure S7. Overexpression of TMPRSS2 in 293T and 293T-A2 cells. (**A-B**) Western blotting analysis of TMPRSS2 levels in 293T and 293T-A2 cells (**A**), and 293T and 293T-A2 cells with transiently transfection of the human *TMPRSS2-HA* tag plasmid (293T-T2 and 293T-A2T2, **B**).

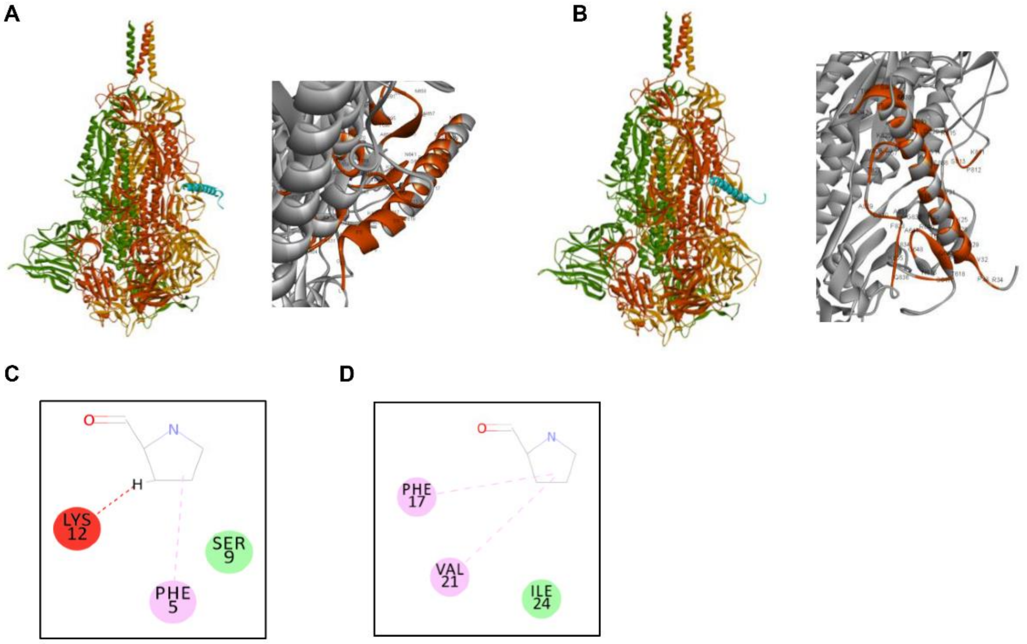

Figure S8. Protein-protein docking analysis of LL-37-SARS-CoV-2 spike. (**A**-**B**) LL-37 was coloured as cyan for Pose 2 (**A**) and Pose 3 (**B**). The binding surface was enlarged and highlighted as red, and the important binding residues were labeled. (**C**-**D**) Calculated 2D interactions of LL-37 in complex with SARS-CoV-2 spike residue of Pro812 for Pose 2 (**C**) and Pose 3 (**D**).

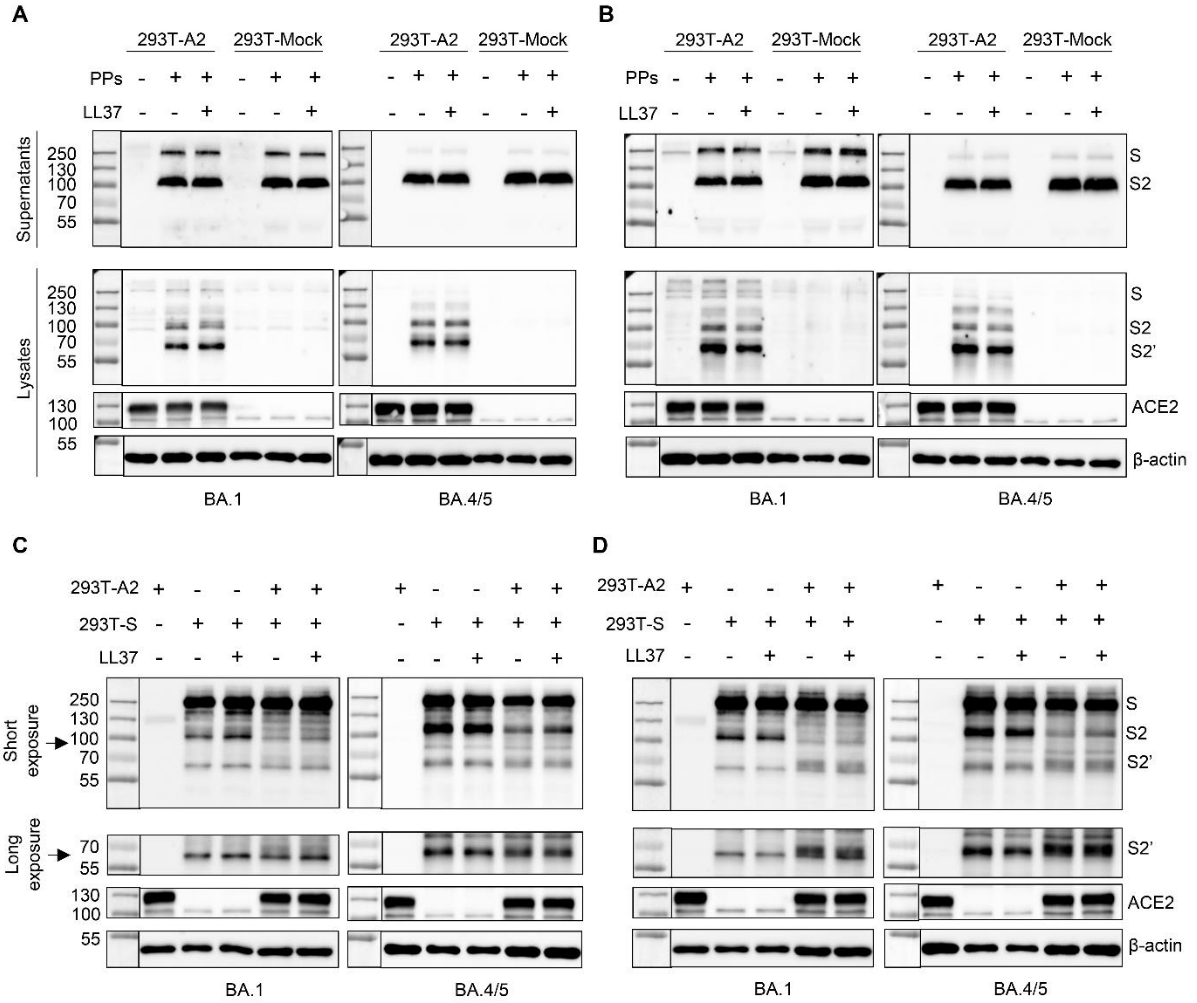

Figure S9. LL-37 did not inhibit the generation of S2' fragment of spike protein of BA.1 and BA.4/5. (**A-B**) Western blotting analysis of S2 subunit and S2' levels in the supernatant and 293T-A2 cells which were co-cultured with pseudoparticles (pretreated for 2 h with or without 10 μg/mL of LL-37) for 2 h (**A**) and 4 h (**B**). 293T cells that transfected with a control vector (293T-Mock) were used as a negative control. PPs: pseudotyped particles. (**C-D**) Western blotting analysis of S2 subunit and S2' levels in the adherent syncytia of the spike-expressing 293T cells (pretreated for 2 h with or without 20 μg/mL of LL-37) and 293T-A2 cells which were co-cultured for 2 h (**C**) and 4 h (**D**). Three bands were captured as: S (~250 kDa), S2 (~100 kDa), and S2' (~68 kDa).

A

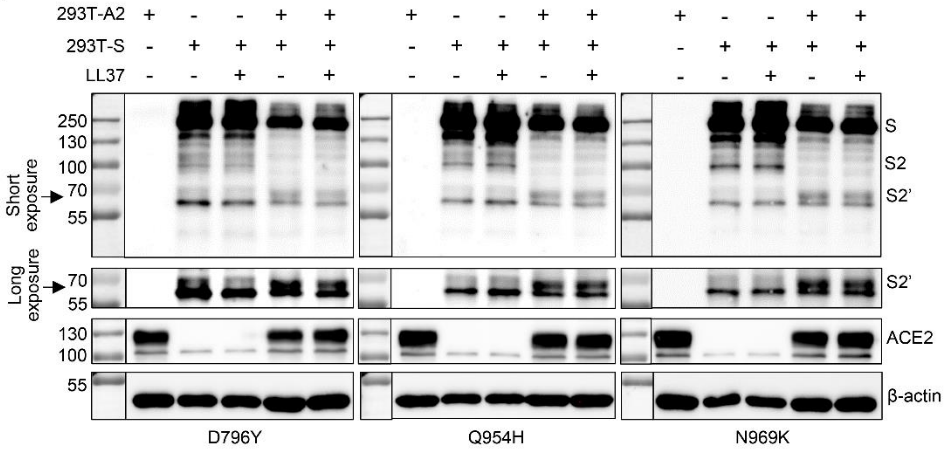

B

Calculate mutation energy of N764K (stability)

Pose	Mutation energy (kcal/mol)	Effect
Pose1	1.37	Destabilizing
Pose2	1.14	Destabilizing
Pose3	1.87	Destabilizing

C

Calculate mutation energy of N764K (binding)

Pose	Mutation energy (kcal/mol)	Effect
Pose1	0.4	Neutral
Pose2	0.39	Neutral
Pose3	0.55	Destabilizing

Figure S10. Western blotting analysis of S2' levels and calculation of mutation energy for folding and binding free energies. (**A**) Western blotting analysis of S2 subunit and S2' levels in the adherent syncytia of the spike-expressing 293T cells (with D796Y, Q954H, or N969K mutation, pretreated for 2 h with or without 20 μg/mL of LL-37) and 293T-A2 cells which were co-cultured for 8 h. Three bands were captured as: S (~250 kDa), S2 (~100 kDa), and S2' (~68 kDa). (**B-C**) The complexes of three poses, *i.e.* Pose 1, Pose 2 and Pose 3 (Fig. 2 and Supplementary Fig. 10), were performed single mutation of N764K in SARS-CoV-2 spike protein, and the mutation energy and corresponding effect were shown as stability (**B**) and binding (**C**).

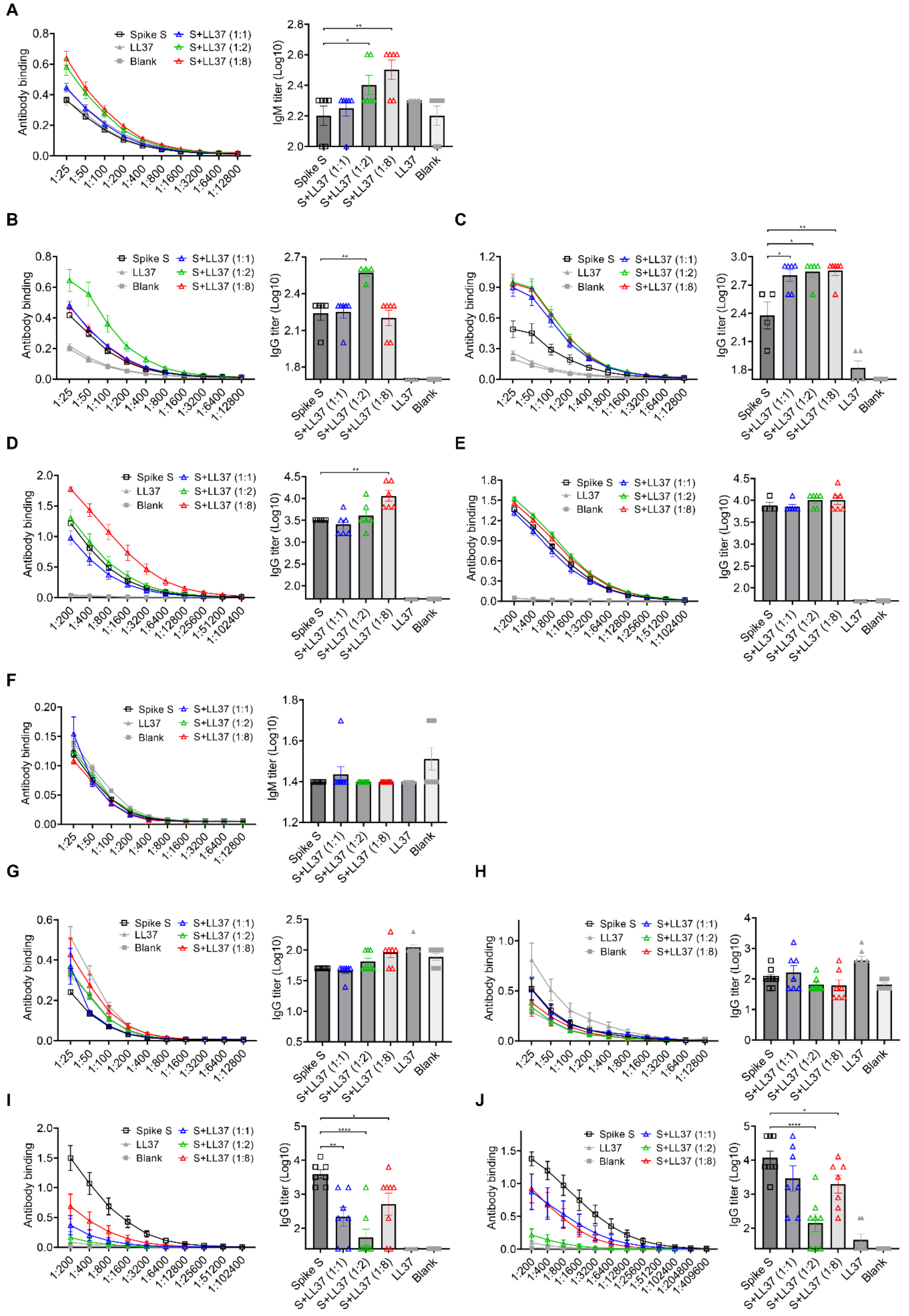

Figure S11. LL-37 enhanced the humoral immune response induced by spike of SARS-CoV-2 but not

the BA.4/5 variant. (**A-E**) Serum antibody response against the SARS-CoV-2 spike protein in mice. ELISA analysis of IgM responses and titers on day 7 (**A**), and IgG responses and titers on day 7 (**B**), day 14 (**C**), day 21 (**D**) and day 28 (**E**) to the SARS-CoV-2 spike protein. (**F-J**) Serum antibody response against the BA.4/5 spike protein in mice. ELISA analysis of IgM responses and titers on day 7 (**F**), and IgG responses and titers on day 7 (**G**), day 14 (**H**), day 21 (**I**) and day 28 (**J**) to the BA.4/5 spike protein. Serum antibody binding was measured as absorbance at 450 nm. Data represent the mean± SEM. Significance is indicated by * $P \leq 0.05$, ** $P \leq 0.01$, **** $P \leq 0.0001$.

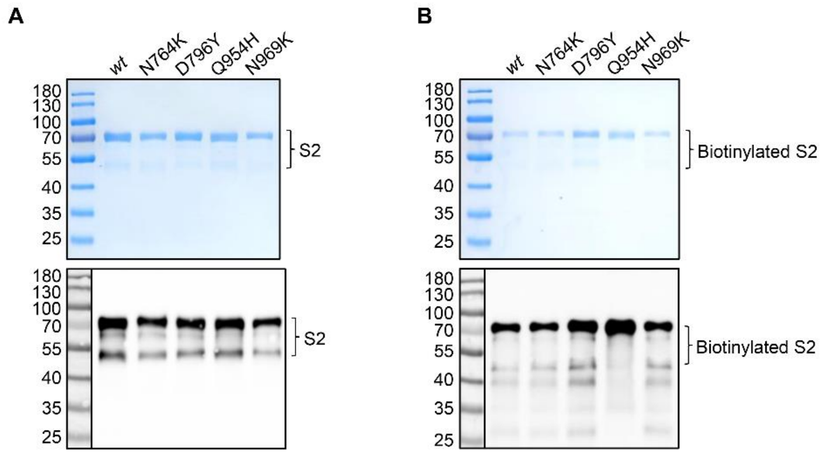

Figure S12. SDS-PAGE analysis for the purified and biotinylated S2 proteins. (**A**) The purity of indicated types of proteins was measured by SDS–PAGE and visualized by staining with FastBlue (upper) and by western blotting (below). (**B**) The biotin labeling efficiency of indicated types of proteins was detected by SDS–PAGE and visualized by staining with FastBlue (upper) and by western blotting using the HRP-labeled streptavidin (below).
